# Penalized Quadratic Inference Function-Based Variable Selection for Generalized Partially Linear Varying Coefficient Models with Longitudinal Data

**DOI:** 10.1155/2020/3505306

**Published:** 2020-10-05

**Authors:** Jinghua Zhang, Liugen Xue

**Affiliations:** ^1^Department of Information Engineering, Jingdezhen Ceramic Institute, Jiangxi, China; ^2^College of Applied Sciences, Beijing University of Technology, Beijing, China

## Abstract

Semiparametric generalized varying coefficient partially linear models with longitudinal data arise in contemporary biology, medicine, and life science. In this paper, we consider a variable selection procedure based on the combination of the basis function approximations and quadratic inference functions with SCAD penalty. The proposed procedure simultaneously selects significant variables in the parametric components and the nonparametric components. With appropriate selection of the tuning parameters, we establish the consistency, sparsity, and asymptotic normality of the resulting estimators. The finite sample performance of the proposed methods is evaluated through extensive simulation studies and a real data analysis.

## 1. Introduction

Identifying the significant variables is of great significance in all regression analysis. In practice, a number of variables are available for an initial analysis, but many of them may not be significant and should be excluded from the final model in order to increase the accuracy of prediction. Various procedures and criteria, such as stepwise selection and subset selection with Akaike information criterion (AIC), Mallows Cp, and Bayesian information criterion (BIC), have been developed. Nevertheless, these selection methods suffer from expensive computational costs. Many shrinkage methods have been developed for the purpose of computational efficiency, e.g., the nonnegative garrote [1], the LASSO [2], the bridge regression [3], the SCAD [4], and the one-step sparse estimator [5]. Among those, the SCAD possesses the virtues of continuity, unbiasedness, and sparsity. There are a number of works on the SCAD estimation methods in various regression models, e.g., [6–9]. Zhao and Xue [8] proposed a variable selection method to select significant variables in the parametric components and the nonparametric components simultaneously for the varying coefficient partially linear models (VCPLMs).

On the other hand, longitudinal data occurs frequently in biology, medicine, and life science, in which it is often necessary to make repeated measurements of subjects over time. The responses from different subjects are independent, but the responses from the same subject are very likely to be correlated. This feature is called “within-cluster correlation”. Qu et al. [10] proposed a method of quadratic inference functions (QIFs) to treat the longitudinal data. The QIF can efficiently take the within-cluster correlation into account and is more efficient than the generalized estimating equation (GEE) [11] approach when the working correlation is misspecified. The QIF approach has been applied to many models, including varying coefficient models (VCM) [12, 13], partially linear models (PLM) [14], varying coefficient partially linear models (VCPLMs) [15], and generalized partially linear models (GPLM) [16]. Wang et al. [13] proposed a group SCAD procedure for variable selection of VCM with longitudinal data. More recently, Tian et al. [15] proposed a QIF-based SCAD penalty for the variable selection for VCPLM with longitudinal data.

As introduced in Li and Liang [17], the generalized partially linear varying coefficient model (GPLVCM) possesses the great flexibility of a nonparametric regression model and provides the explanatory power of a generalized linear regression model, which arises naturally due to categorical covariates. Many models are the special case of GPLVCM, e.g., VCM, VCPLM, PLM, and GLM. Li and Liang [17] studied variable selection for GPLVCM, where the parametric components are identified via the SCAD but the nonparametric components are selected via a generalized likelihood ratio test instead of shrinkage. In this paper, we extend the QIF-based group SCAD variable selection procedure to GPLVCM with longitudinal data, and the B-spline methods are adopted to approximate the nonparametric component in the model. With suitable chosen tuning parameters, the proposed variable selection procedure is consistent, and the estimators of regression coefficients have oracle property, i.e., the estimators of the nonparametric components achieve the optimal convergence rate, and the estimators of the parametric components have the same asymptotic distribution as that based on the correct submodel.

The rest of this paper is organized as follows. In Section 2, we propose a variable selection procedure for the GPLVCM with longitudinal data. Asymptotic properties of the resulting estimators and an iteration algorithm are presented in Section 3. In Section 4, we carry out simulation studies to assess the finite sample performance of the method. A real data analysis is given in Section 5 to illustrate the proposed methodology. The details of proofs are provided in the appendix.

## 2. Methodology

### 2.1. GPLVCM with Longitudinal Data

In this article, we consider a longitudinal study with *n* subjects and *m*_*i*_ observations over time for the *i*th subject (*i* = 1, ⋯, *n*) for a total of *N* = ∑_*i*=1_^*n*^ *m*_*i*_ observations. Each observation consists of a response variable *Y*_*ij*_ and the predicator variables (*X*_*ij*_, *Z*_*ij*_, *U*(*ij*)), where *X*_*ij*_ ∈ *R*^*p*^, *Z*_*ij*_ ∈ *R*^*q*^ and *U*_*ij*_ is a scalar. We assume that the observations from different subjects are independent, but those within the same subject are dependent. The generalized varying coefficient partially linear model (GPLVM) with longitudinal data takes the form

(1) $$\begin{array}{c} \mu_{ij}=E\left(Y_{ij}\;|\;X_{ij},Z_{ij},U_{ij}\right)=h\left(X_{ij}^{T}\beta +Z_{ij}^{T}\alpha \left(U_{ij}\right)\right), \end{array}$$

where *μ*_*ij*_ is the expectation of *Y*_*ij*_ when *X*_*ij*_, *Z*_*ij*_, and *U*_*ij*_ are given, *β* = (*β*_1_,⋯,*β*_*p*_)^*T*^ is an unknown *p* × 1 regression coefficient vector, *h*(·) is a known smooth link function, and *α*(*u*) = (*α*_1_(*u*), *α*_2_(*u*),⋯,*α*_*q*_(*u*))^*T*^ is a *q* × 1 unknown monotonic smooth function vector. Without loss of generality, we assume *U* ~ *U*[0, 1].

We approximate *α*(·) by B-spline basis functions *B*(*u*) = (*B*_1_(*u*),⋯,*B*_*L*_(*u*))^*T*^ with the order of *M*, where *L* = *K* + *M* + 1 and *K* is the number of interior knots, i.e.,

(2) $$\begin{array}{c} \alpha_{k}\left(u\right)\approx \alpha_{k}^{*}\left(u\right)=B{\left(u\right)}^{T}\gamma_{k},\;k=1,\cdots ,q, \end{array}$$

where *γ*_*k*_ = (*γ*_*k*1_,⋯,*γ*_*kL*_)^*T*^ is a *L* × 1 vector of unknown regression coefficients. Accordingly, *μ*_*ij*_ is approximated by

(3) $$\begin{array}{c} \mu_{ij}=E\left(Y_{ij}\;|\;X_{ij},Z_{ij},U_{ij}\right)=h\left(X_{ij}^{T}\beta +Z_{ij}^{T}\cdot I_{q}⊗B{\left(U_{ij}\right)}^{T}\gamma \right), \end{array}$$

where *γ* = (*γ*_1_^*T*^, ⋯,*γ*_*q*_^*T*^)^*T*^ and “⊗” is the Kronecker product. We use the B-spline basis functions because they are numerically stable and have bounded support [18]. The spline approach also treats a nonparametric function as a linear function with the basis functions as pseudodesign variables, and thus, any computational algorithm for the generalized linear models can be used for the GPLVCMs.

To incorporate the within-cluster correlation, we apply the QIFs to estimate *β* and *γ*, respectively. Denote *θ* = (*β*^*T*^, *γ*^*T*^)^*T*^, we define the extended score *g*_*N*_(*θ*) as follows:

(4) $$\begin{array}{c} g_{N}\left(\theta \right)=\frac{1}{n}\overset{n}{\underset{i=1}{\sum }}\;g_{i}\left(\theta \right)=\frac{1}{n}\overset{n}{\underset{i=1}{\sum }}\;\left(\begin{array}{c} {\dot{\mu }}_{i}^{T}A_{i}^{-\frac{1}{2}}M_{1}A_{i}^{-\frac{1}{2}}\left(Y_{i}-\mu_{i}\right)\\ \vdots \\ {\dot{\mu }}_{i}^{T}A_{i}^{-\frac{1}{2}}M_{s}A_{i}^{-\frac{1}{2}}\left(Y_{i}-\mu_{i}\right) \end{array}\right), \end{array}$$

where ${\dot{\mu }}_{i}=\partial \mu_{i}/\partial \theta$, *A*_*i*_ = diag(Var(*Y*_*i*1_), ⋯, Var(*Y*_*im*_)) is the marginal variance matrix of subject *Y*_*i*_, and *M*_1_, ⋯, *M*_*s*_ are the base matrices to represent the inverse of the working correlation matrix *R* in GEE approach. Following Qu et al. [10], we define the quadratic inference functions to be

(5) $$\begin{array}{c} Q_{n}\left(\theta \right)=ng_{N}^{T}\left(\theta \right)\Omega_{n}{\left(\theta \right)}^{-1}g_{N}\left(\theta \right), \end{array}$$

where *Ω*_*n*_(*θ*) = (1/*n*)∑_*i*=1_^*n*^ *g*_*i*_(*θ*)*g*_*i*_(*θ*)^*T*^. Note that *Ω*_*n*_ depends on *θ*. The QIF estimate $\tilde{\theta }$ is then given by

(6) $$\begin{array}{c} \tilde{\theta }=\text{argmi}{\text{n}}_{\theta }Q_{n}\left(\theta \right). \end{array}$$

### 2.2. Penalized QIF

In real data analysis, the true regression model is always unknown. An overfitted model lowers the efficiency of estimation while an underfitted one leads to a biased estimator. A popular approach to identify the relevant predictors while estimating the nonzero parameters and functions in model (1) simultaneously is to exert some kind of “penalty” on the original objective function. Here, we choose the smoothly clipped absolute deviation (SCAD) penalty because it has several advantages such as unbiasedness, sparsity, and continuity. The SCAD-penalized quadratic inference function (PQIF) is defined as follows:

(7) $$\begin{array}{c} Q_{n}^{p}\left(\theta \right)=Q_{n}\left(\theta \right)+n\overset{p}{\underset{l=1}{\sum }}\;p_{\lambda_{1}}\left(\left|\beta_{l}\right|\right)+n\overset{q}{\underset{k=1}{\sum }}\;p_{\lambda_{2}}\left({\left\|\gamma_{k}\right\|}_{H}\right), \end{array}$$

where ‖*γ*_*k*_‖_*H*_ = (*γ*_*k*_^*T*^*Hγ*_*k*_)^1/2^, *H* = (*h*_*ij*_)_*L*×*L*_, *h*_*ij*_ = ∫_0_^1^ *B*_*i*_(*u*)*B*_*j*_^*T*^(*u*)*du* and *p*_*λ*_ is the SCAD penalty function, where the derivative is defined as

(8) $$\begin{array}{c} p\prime_{l}\left(\omega \right)=\lambda \left\{I\left(\omega ⩽l\right)+\frac{{\left(a\lambda -\omega \right)}_{+}}{\left(a-1\right)\lambda }I\left\{\omega >\lambda \right\}\right\}, \end{array}$$

where *a* > 2, *ω* > 0, *p*_*λ*_(0) = 0; here, we choose *a* = 3.7 as in [4].

Note that

(9) $$\begin{array}{c} {\left\|\gamma_{k}\right\|}_{H}={\left(\int_{0}^{1}\;\gamma_{k}^{T}B\left(u\right)B^{T}\left(U\right)\gamma_{k}du\right)}^{1/2}={\left(\int_{0}^{1}\;{\left[\alpha^{*}\left(u\right)\right]}^{2}du\right)}^{1/2}. \end{array}$$

This group-wised penalization ensures that the spline coefficient vector of the same nonparametric component is treated as an entire group in model selection.

Denote $\hat{\theta }$ to be the penalized estimator obtained by minimizing the penalized objective function of (7). Then, $\hat{\beta }={\left({\overset{\wedge }{\theta }}_{1},\cdots ,{\overset{\wedge }{\theta }}_{p}\right)}^{T}$ is the estimator of the parameter *β* and the estimator of the nonparametric function *α*(*u*) is calculated by $\hat{\alpha }\left(u\right)=B{\left(u\right)}^{T}\hat{\gamma }$, where $\hat{\gamma }={\left({\overset{\wedge }{\gamma }}_{1}^{T},\cdots ,{\overset{\wedge }{\gamma }}_{q}^{T}\right)}^{T}={\left({\overset{\wedge }{\theta }}_{p+1},\cdots ,{\overset{\wedge }{\theta }}_{p+L},{\overset{\wedge }{\theta }}_{p+L+1},\cdots ,{\overset{\wedge }{\theta }}_{p+qL}\right)}^{T}$.

## 3. Asymptotic Properties

### 3.1. Oracle Property

We next establish the asymptotic properties of the resulting penalized QIF estimators. We first introduce some notations. Let *β*_0_ and *α*_0_(·) denote the true values of *β*(·) and *α*(·). In addition, *γ*_0_ is the spline coefficient vector from the spline approximation to *α*_0_(·). Without loss of generality, we assume that *β*_0*l*_ ≠ 0, *l* = 1, ⋯, *p*_1_ and *β*_0*l*_ = 0, *l* = *p*_1_ + 1, ⋯, *p*, i.e., only the first *p*_1_ component of *β*_0_ is nonzero. Similarly, we assume that *α*_0*k*_(·) ≠ 0, *k* = 1, ⋯, *q*_1_ and *α*_0*k*_(·) = 0, *k* = *q*_1_ + 1, ⋯, *q*, i.e., only the first *q*_1_ component of *α*_0_(·) is nonzero. For convenience and simplicity, let *C* denote a positive constant that may have different values at each appearance throughout this paper and ||*A*|| denote the modulus of the largest singular value of matrix or vector *A*. Before the proof of our main theorems, we list some regularity conditions used in this paper.

**Assumption 1 (A1).**

The spline regression parameter *γ* is identifiable, that is, *γ*_0_ is the spline coefficient vector from the spline approximation to *α*_0_(·). In addition, there is a unique *θ*_0_ = (*β*_0_, *γ*_0_) ∈ *S* satisfying *E*{*g*_*N*_(*θ*_0_)} = 0, where *S* is the parameter space.

**Assumption 2 (A2).**

The weight matrix *Ω*_*n*_ = (1/*n*)∑_*i*=1_^*n*^ *g*_*i*_(*θ*)*g*_*i*_^*T*^(*θ*) converges almost surely to a constant matrix *Ω*_0_, where *Ω*_0_ is invertible.

**Assumption 3 (A3).**

The covariate matrices *X*_*i*_ and *Z*_*i*_, *i* = 1, ⋯, *n*, satisfy sup_*i*_*E*‖*X*_*i*_‖^4^ < ∞ and sup_*i*_*E*‖*Z*_*i*_‖^4^ < ∞.

**Assumption 4 (A4).**

The error *ε*_*i*_ = *Y*_*i*_ − *μ*_*i*_ satisfies *E*(*ε*_*i*_*ε*_*i*_^*T*^) = *V*_*i*_, sup_*i*_‖*V*_*i*_‖ < ∞, and there exists a positive constant *δ* such that sup_*i*_*E*‖*ε*_*i*_‖^2+*δ*^ < ∞.

**Assumption 5 (A5).**

All marginal variances *A*_*i*_ ≥ 0 and sup_*i*_‖*A*_*i*_‖ < ∞.

**Assumption 6 (A6).**

{*m*_*i*_} is a bounded sequence of positive integers.

**Assumption 7 (A7).**

*α*_*i*_(*u*), *i* = 1, 2, ⋯, *q* is *r*th continuous differentiable on (0, 1), where *r* ≥ 2.

**Assumption 8 (A8).**

The inner knots {*c*_*i*_, *i* = 1, ⋯, *K*} satisfy

(10) $$\begin{array}{c} \underset{1\leq i\leq K}{\max}\left|h_{i+1}-h_{i}\right|=o\left(K^{-1}\right),\\ \frac{\max h_{i}}{\min h_{i}}\leq C_{0}, \end{array}$$

where *h*_*i*_ = *c*_*i*_ − *c*_*i*−1_.

**Assumption 9 (A9).**

The link function *h*(·) is 2th continuous differentiable and *E*{*h*^2+*δ*^} < ∞ for some *δ* > 2.

**Assumption 10 (A10).**

*a*_*n*_ = *O*(*n*^−1/2^); *b*_*n*_⟶0 as *n*⟶∞, where

(11) $$\begin{array}{c} a_{n}=\underset{k,l}{\max}\left\{\left|p\prime_{\lambda_{1}}\left(\left|\beta_{0l}\right|\right)\right|,\left|p\prime_{\lambda_{2}}\left({\left\|\gamma_{0k}\right\|}_{H}\right)\right|\text{: }\beta_{ol}\neq 0,\gamma_{0k}\neq 0\right\},\\ b_{n}=\underset{k,l}{\max}\left\{\left|p\prime \prime_{\lambda_{1}}\left(\left|\beta_{0l}\right|\right)\right|,\left|p\prime \prime_{\lambda_{2}}\left({\left\|\gamma_{0k}\right\|}_{H}\right)\right|\text{: }\beta_{ol}\neq 0,\gamma_{0k}\neq 0\right\}. \end{array}$$

Theorem 1 indicates that the estimator of nonparametric components achieve the optimal convergence rate.

**Theorem 1 .**

Assume that Assumptions (A.1)–(A.10) hold and the number of knots *K* = *O*(*N*^1/(2*r* + 1)^), then

(12) $$\begin{array}{c} \left\|{\hat{\alpha }}_{k}\left(\cdot \right)-\alpha_{0k}\left(\cdot \right)\right\|=O_{p}\left(n^{-r/\left(2r+1\right)}\right),\;\;k=1,\cdots ,q. \end{array}$$

Furthermore, under suitable condition, Theorem 1 shows that the penalized QIF estimator has the sparsity property.

**Theorem 2 .**

Assume that the conditions in Theorem 1 hold and $\lambda_{\max}⟶0,\sqrt{n}\lambda_{\min}⟶\infty$ as *n*⟶∞, with probability approaching 1,

(13) $$\begin{array}{c} {\hat{\beta }}_{l}=0,\;l=p_{1}+1,\cdots ,p,\\ {\hat{\alpha }}_{k}\left(\cdot \right)\equiv 0,\;k=q_{1}+1,\cdots ,q, \end{array}$$

where *λ*_max_ = max{*λ*_1_, *λ*_2_}, *λ*_min_ = min{*λ*_1_, *λ*_2_}.

Theorems 1 and 2 indicate that with the tune parameter *λ* being suitably chosen, the proposed selection method possesses model selection consistency. Next, we establish the asymptotic property for the estimator of the nonzero parametric components. Let *β*^∗^ = (*β*_1_, ⋯,*β*_*p*_1__)^*T*^, *α*^∗^(·) = (*α*_1_^∗^(·), ⋯,*α*_*q*_1__^∗^(·))^*T*^ and let *β*_0_^∗^ and *α*_0_^∗^(·) denote their true value, respectively. In addition, let *γ*^∗^ = (*γ*_1_^*T*^, ⋯,*γ*_*q*_1__^*T*^)^*T*^ and *γ*_0_^∗^ = (*γ*_01_^*T*^, ⋯,*γ*_0*q*_1__^*T*^)^*T*^ denote the spline coefficient vector of *α*^∗^(·) and *α*_0_^∗^(·), respectively, and let *X*_*i*_^∗^ and *Z*_*i*_^∗^, *i* = 1, ⋯, *n* denote their correspondent covariate. Let ${\tilde{X}}_{i}=H^{\prime }\left(\eta_{i}\right)X_{i}^{*},\tilde{X}=\left({\tilde{X}}_{1}^{T},\cdots ,{\tilde{X}}_{n}^{T}\right),{\tilde{W}}_{i}=H^{\prime }\left(\eta_{i}\right)W_{i}^{*},\tilde{W}=\left({\tilde{W}}_{1}^{T},\cdots ,{\tilde{W}}_{n}^{T}\right)$, and

(14) $$\begin{array}{c} \Gamma =E\left\{{\tilde{X}}^{T}\tau \tilde{X}-E\left[{\tilde{X}}^{T}\tau \tilde{W}\left|u\right.\right]E{\left[{\tilde{W}}^{T}\tau \tilde{W}\left|u\right.\right]}^{-1}{\tilde{W}}^{T}\tau \tilde{X}\right\},\\ \Delta =E{\left\{\left[\tau -E\left[{\tilde{X}}^{T}\tau \tilde{W}\left|u\right.\right]E{\left[{\tilde{W}}^{T}\tau \tilde{W}\left|u\right.\right]}^{-1}E\left[{\tilde{W}}^{T}\tau \left|u\right.\right]\right]\varepsilon \right\}}^{⊗2}, \end{array}$$

where Δ^⊗2^ = ΔΔ^*T*^, *τ* = (*τ*_*ij*_)_*n*×*n*_ is a *n* × *n* block matrix with its (*i*, *j*) block taking the form

(15) $$\begin{array}{c} \tau_{ij}=\overset{s}{\underset{k=1}{\sum }}\;\overset{s}{\underset{l=1}{\sum }}\;A_{i}^{-1/2}M_{k}A_{i}^{-1/2}H^{\prime }\left(\eta_{i}\right)P_{i}^{*}\Omega_{lk}^{-1}P_{j}^{*T}H^{\prime }\left(\eta_{j}\right)A_{j}^{-1/2}M_{l}A_{j}^{-1/2}. \end{array}$$

Theorem 3 states that ${\overset{\wedge }{\beta }}^{*}$ is asymptotically normally distributed.

**Theorem 3 .**

Suppose that Assumptions (A.1)–(A.9) hold and the number of knots *K* = *O*(*N*^1/(2*r* + 1)^), then

(16) $$\begin{array}{c} \sqrt{n}\left({\overset{\wedge }{\beta }}^{*}-\beta_{0}^{*}\right)\overset{L}{⟶}N\left(0,\Sigma \right), \end{array}$$

where *Σ* = (ΓΔ^−1^Γ)^−1^ and $\overset{L}{⟶}$ represents the convergence in distribution.

### 3.2. Selection of Tuning Parameters

Theorems 1–3 imply that the proposed variable selection procedure possessed the oracle property. However, this attractive feature relies on the choice of tuning parameters *λ*_*i*_. The popular criteria to choose *λ*_*i*_ include cross-validation, generalized cross-validation, AIC, and BIC. Wang et al. [19] suggested using BIC for the SCAD estimator in linear models and partially linear models and proved its model selection consistency property, i.e., the optimal parameter chosen by BIC can identify the true model with probability tending to one. Tian proved that for partially linear models. Hence, we adopt BIC to choose the optimal {*λ*_1_, *λ*_2_}. Following [19–21], we simplify the tuning parameters as

(17) $$\begin{array}{c} \lambda_{1}=\frac{\lambda_{0}}{{\left\|{\tilde{\gamma }}_{k}^{\left(0\right)}\right\|}_{H}},\\ \lambda_{2}=\frac{\lambda_{0}}{\left|{\tilde{\beta }}_{k}^{\left(0\right)}\right|}, \end{array}$$

where ${\tilde{\beta }}_{k}^{\left(0\right)}$ and ${\tilde{\gamma }}_{k}^{\left(0\right)}$ are the unpenalized QIF estimates. Consequently, the original two-dimensional problem becomes a univariate problem about *λ*_0_, which can be selected according to the following BIC-type criterion:

(18) $$\begin{array}{c} \text{BI}{\text{C}}_{\lambda }=Q_{n}^{p}\left({\hat{\theta }}_{\lambda }\right)+df_{\lambda }\times \log\left(n\right), \end{array}$$

where ${\hat{\theta }}_{\lambda }=\left({\hat{\beta }}_{\lambda },{\hat{\gamma }}_{1\lambda }^{T},\cdots ,{\hat{\gamma }}_{q\lambda }^{T}\right)$ is the regression coefficient estimated by minimizing the penalized QIF in (2.8) for a given *λ* and *df*_*λ*_ is the number of nonzero coefficients of ${\hat{\beta }}_{\lambda }$ and ${\left\|{\hat{\gamma }}_{1\lambda }\right\|}_{H},\cdots ,{\left\|{\hat{\gamma }}_{q\lambda }\right\|}_{H}$. Thus, the tuning parameter *λ* is obtained by

(19) $$\begin{array}{c} \hat{\lambda }=\arg\underset{\lambda }{\min}\text{BI}{\text{C}}_{\lambda }. \end{array}$$

From Theorem 4 of Tian et al. [15], the BIC tuning parameter selector enables us to select the true model consistently.

### 3.3. An Algorithm Using Local Quadratic Approximation

Based on Fan and Li's local quadratic approximating approach [4], we propose an iterative algorithm to minimize the PQIF (7). Similar with Tian et al. [15], we choose the unpenalized QIF estimator $\tilde{\theta }$ as the initial estimator. Let **θ**^*k*^ = (*β*_1_^*k*^, ⋯,*β*_*p*_^*k*^, **γ**_1_^*kT*^, ⋯,**γ**_*q*_^*kT*^)^*T*^ be the value of **θ** at the *k*th iteration. If *β*_*l*_^*k*^ (or **γ**_*l*_^*k*^) is close to 0 (or 0), i.e., |*β*_*l*_^*k*^| ⩽ *ϵ* (or ‖**γ**_*l*_^*k*^‖_*H*_ ⩽ *ϵ*) with some small threshold value *ϵ*, then we set *β*_*l*_^*k*^ = 0 (or **γ**_*l*_^*k*^ = 0). We consider *ϵ* = 10^−6^ in our simulations.

Suppose *β*_*l*_^*k*+1^ = 0, for *l* = *p*_*k*_ + 1, ⋯, *p*, and **γ**_*l*_^*k*+1^ = 0, for *l* = *q*_*k*_ + 1, ⋯, *q*, and **β**^*k*+1^ = (*β*_1_^*k*+1^, ⋯,*β*_*p*_*k*__^*k*+1^, *β*_*p*_*k*_+1_^*k*+1^, ⋯,*β*_*p*_^*k*+1^)^*T*^ = ((**β**_*N*_^*k*+1^)^*T*^, (**β**_*Z*_^*k*+1^)^*T*^)^*T*^, where **β**_*N*_^*k*+1^ = (*β*_1_^*k*+1^, ⋯,*β*_*p*_*k*__^*k*+1^)^*T*^ are the nonzero parametric components and **β**_*Z*_^*k*+1^ = (*β*_*p*_*k*_+1_^*k*+1^, ⋯,*β*_*p*_^*k*+1^)^*T*^ = 0. Similarly, let **γ**^*k*+1^ = ((**γ**_1_^*k*+1^)^*T*^, ⋯,(**γ**_*q*_*k*__^*k*+1^)^*T*^, (**γ**_*q*_*k*_+1_^*k*+1^)^*T*^, ⋯,(**γ**_*q*_^*k*+1^)^*T*^)^*T*^ = ((**γ**_*N*_^*k*+1^)^*T*^, (**γ**_*Z*_^*k*+1^)^*T*^)^*T*^, where **γ**_*N*_^*k*+1^ = ((**γ**_1_^*k*+1^)^*T*^, ⋯,(**γ**_*q*_*k*__^*k*+1^)^*T*^)^*T*^ and **γ**_*Z*_^*k*+1^ = ((**γ**_*q*_*k*_+1_^*k*+1^)^*T*^, ⋯,(**γ**_*q*_^*k*+1^)^*T*^)^*T*^ correspond to *q*_*k*_ zero functions and *q* − *q*_*K*_ zero functions, respectively. Let **θ** = (**β**_*N*_^*T*^, **β**_*Z*_^*T*^, **γ**_*N*_^*T*^, **γ**_*Z*_^*T*^)^*T*^ denote a vector which has the same length and same partition with **θ**^*k*+1^.

For the parametric term, if |*β*_*l*_^*k*^| > *ϵ*, the penalty function at *β*_*l*_ ≈ *β*_*l*_^*k*^ is approximated by

(20) $$\begin{array}{c} p_{\lambda }\left(\beta_{l}\right)\approx p_{\lambda }\left(\beta_{l}^{k}\right)+\frac{1}{2}\frac{p_{\lambda }^{\prime }\left(\left|\beta_{l}^{k}\right|\right)}{\left|\beta_{l}^{k}\right|}\left(\beta_{l}^{2}-{\left(\beta_{l}^{k}\right)}^{2}\right). \end{array}$$

Similarly, to the nonparametric component, if ‖**γ**_*l*_^*k*^‖_*H*_ > *ϵ*, the penalty function at **γ**_*l*_ ≈ **γ**_*l*_^*k*^ is approximated by

(21) $$\begin{array}{c} p_{\lambda }\left({\left\|\gamma_{l}\right\|}_{H}\right)\approx p_{\lambda }\left({\left\|\gamma_{l}^{k}\right\|}_{H}\right)+\frac{1}{2}\frac{p_{\lambda }^{\prime }\left({\left\|\gamma_{l}^{k}\right\|}_{H}\right)}{{\left\|\gamma_{l}^{k}\right\|}_{H}}\left({{\left\|\gamma_{l}\right\|}_{H}}^{2}-{{\left\|\gamma_{l}^{k}\right\|}_{H}}^{2}\right)=p_{\lambda }\left({\left\|\gamma_{l}^{k}\right\|}_{H}\right)+\frac{1}{2}\frac{p\prime_{\lambda }\left({\left\|\gamma_{l}^{k}\right\|}_{H}\right)}{{\left\|\gamma_{l}^{k}\right\|}_{H}}\left(\beta_{l}^{T}H\beta_{l}-\beta_{l}^{kT}H\beta_{l}^{k}\right), \end{array}$$

where *p*′_*λ*_ is the first-order derivative of the penalty function *p*_*λ*_. This leads to the local approximation of the PQIF 𝒬_*n*_^*p*^(**θ**) by a quadratic function:

(22) $$\begin{array}{c} Q_{n}\left(\theta^{k}\right)+{\dot{Q}}_{n}{\left(\theta^{k}\right)}^{T}\left(\omega_{11}-\omega_{11}^{k}\right)+\frac{1}{2}{\left(\omega_{11}-\omega_{11}^{k}\right)}^{T}{\ddot{Q}}_{n}\left(\theta^{k}\right)\left(\omega_{11}-\omega_{11}^{k}\right)+\frac{n}{2}\omega_{11}^{t}\Lambda \left(\theta^{k}\right)\omega_{11}, \end{array}$$

where ${\dot{𝒬}}_{n}\left(\theta^{k}\right)=\partial {𝒬}_{n}\left(\theta^{k}\right)/\partial \omega_{11}$, $\;{\ddot{𝒬}}_{n}\left(\theta^{k}\right)=\partial^{2}{𝒬}_{n}\left(\theta^{k}\right)/\partial \omega_{11}\partial \omega_{11}^{T}$, with **ω**_11_ = (**β**_*N*_^*T*^, **γ**_*Z*_^*T*^)^*T*^, and

(23) $$\begin{array}{c} \Lambda \left(\theta^{k}\right)=\mathrm{diag}\;\left\{\frac{p\prime_{\lambda_{2}}\left(\left|\beta_{1}^{k}\right|\right)}{\left|\beta_{1}^{k}\right|},\cdots ,\frac{p\prime_{\lambda_{2}}\left(\beta_{p_{k}}^{k}\right)}{\left|\beta_{p_{k}}^{k}\right|},\frac{p\prime_{\lambda_{1}}\left({\left\|\gamma_{1}^{k}\right\|}_{H}\right)}{{\left\|\gamma_{1}^{k}\right\|}_{H}}H,\cdots ,\frac{p\prime_{\lambda_{1}}\left({\left\|\gamma_{q_{k}}^{k}\right\|}_{H}\right)}{{\left\|\gamma_{q_{k}}^{k}\right\|}_{H}}H\right\}. \end{array}$$

Minimizing the quadratic function (22), we obtain **ω**_11_^*k*+1^. The Newton-Raphson method then iterates the following process to convergence:

(24) $$\begin{array}{c} \omega_{11}^{k+1}=\omega_{11}^{k}-{\left\{{\ddot{Q}}_{n}\left(\omega_{11}^{k}\right)+n\Lambda \left(\omega_{11}^{k}\right)\right\}}^{-1}\left\{{\dot{Q}}_{n}\left(\omega_{11}^{k}\right)+n\Lambda \left(\omega_{11}^{k}\right)\omega_{11}^{k}\right\}. \end{array}$$

## 4. Simulation Studies

### 4.1. Assessing Rule

In this section, we conduct a simulation study to assess the finite sample performance of the proposed procedures. Following [17], the performance of estimator $\hat{\beta }$ will be assessed by the generalized mean square error (GMSE), which is defined as

(25) $$\begin{array}{c} \text{GMSE}=\frac{1}{n}\overset{n}{\underset{i=1}{\sum }}\;\left(\hat{\beta }-\beta \right)X_{i}^{*}{X_{i}^{*}}^{T}\left(\hat{\beta }-\beta \right). \end{array}$$

The performance of estimator $\hat{\alpha }\left(\cdot \right)$ will be assessed by the square root of average square errors (RASE)

(26) $$\begin{array}{c} \text{RASE}={\left\{\frac{1}{M}\overset{M}{\underset{v=1}{\sum }}\;\overset{q}{\underset{k=1}{\sum }}\;{\left[{\overset{\wedge }{\alpha }}_{k}\left(u_{v}\right)-\alpha_{k}\left(u_{v}\right)\right]}^{2}\right\}}^{1/2}, \end{array}$$

where *u*_*v*_, *v* = 1, ⋯, *M* are the grid points where the function $\hat{\alpha }\left(u\right)$ is evaluated. In our simulation, *M* = 300 is used.

To assess the performance of the variable selection, we use “*C*” to denote the average number of zero regression coefficients that are correctly estimated as zero and use “IC” to denote the average number of nonzero regression coefficients that are erroneously set to zero. The more closer the value of “*C*” to the number of true zero coefficient in the model and the more closer the value of “IC” to zero, the better the performance of the variable selection procedure is.

In our simulations, we use the sample quantiles of *U*_*ij*_ as knots and take the number of internal knots to be 3, that is, *O*(*N*^1/5^). This particular choice is consistent with the asymptotic theory in Section 3 and performs well in the simulations. For each simulated dataset, the proposed estimation procedures for finding out penalized QIF estimators with SCAD and LASSO penalty functions are considered. The tuning parameters *λ*_1_, *λ*_2_ for the penalty functions are chosen by BIC from 50 equispaced grid points in [−15, 5]. For each of these methods, the average of zero coefficients over the 500 simulated datasets is reported.

### 4.2. Study 1 (Partial Penalty)

Consider a Bernoulli response

(27) $$\begin{array}{c} \text{logit}\left\{Y_{ij}\right\}=X_{ij}^{T}\beta +\alpha \left(U_{ij}\right), \end{array}$$

where *β* = (2,1.5,0.7, 0_17_^*T*^)^*T*^, *m* = 6, *X*_*ij*_ ~ *N*(0, *I*_20_), *α*(*U*_*ij*_) = 0.4cos((*π*/2)*U*_*ij*_), and *U*_*ij*_ are drawn independently from *U*[0, 1]. Response variable *Y*_*ij*_ with compound symmetry correlation structure (CS) is generated according to Oman [22]. In our simulation study, we consider *ρ* = 0.25 and 0.75, representing weak and strong correlations, respectively. In some situations, we prefer not to shrink some certain components in the variable selection procedure when some kind of prior information is available. Partial penalty arises naturally for such case. In this example, we only exert penalty on the parametric component, i.e., coefficient *β*. In this situation, the PQIF (7) becomes

(28) $$\begin{array}{c} Q_{n}^{p}\left(\theta \right)=Q_{n}\left(\theta \right)+n\overset{p}{\underset{l=1}{\sum }}\;p_{\lambda_{1}}\left(\beta_{l}\right). \end{array}$$

The variable selection result is reported in Tables 1 and 2.

Tables 1 and 2 show that the performance of the proposed variable selection approach improves as *n* increases, e.g., the number of correctly recognized zero coefficient increases to the number of true zero coefficient in the model and the GMSE of $\hat{\beta }$ decreases as *n* increases. In addition, the RASE of $\hat{\alpha }\left(u\right)$ also decreases as *n* increases, which means the estimated curve of $\hat{\alpha }\left(u\right)$ fits better to the true line of *α*(*u*) when the sample size increases. Moreover, the SCAD penalty method outperforms the LASSO penalty ones in the sense of correct variable selection rate, which significantly reduces the model uncertainty and complexity.

### 4.3. Study 2 (Fixed-Dimensional Setup)

In this example, we generate data from the following model:

(29) $$\begin{array}{c} \text{logit}\left\{Y_{ij}=1\;|\;X_{ij},U_{ij}\right\}=X_{ij}^{T}\beta +Z_{ij}^{T}\alpha \left(U_{ij}\right), \end{array}$$

where *β* = (2,1.5,0.7, 0_7_^*T*^) and *α*(*u*) = (*α*_1_(*u*), *α*_2_(*u*), 0_5_^*T*^)^*T*^ with *α*_1_(*u*) = 0.8cos((*π*/2)*u*), *α*_2_(*u*) = 1.5 + *u*^2^, *X*_*ij*_ and *Z*_*ij*_(*j* = 1, ⋯, 6) come from a multivariate normal distribution with mean zero, marginal variance 1 and correlation coefficient 0.5, and *u* ~ *U*(0, 1). Response variable *Y*_*ij*_ with compound symmetry correlation structure (CS) is generated by the same method as study 1 and we also consider *ρ* = 0.25 and 0.75, representing weak and strong correlations, respectively. We generated 500 datasets for each pair of (*N*, *ρ*). The results are also reported in Tables 3 and 4.

Table 3 reports the variable selection for the parametric components; it shows that the performances become better and better as *n* increases, e.g., the number of correctly recognized zero coefficients, which is denoted as values in the column labeled “*C*,” becomes more and more closer to the true number of zero regression coefficients in the model. At the same time, the GMSE decreases steadily as *n* increases.  Table 4 shows that, for the nonparametric components, the performances of the proposed variable selection method are similar to those of the method for the parametric components. As *n* increases, the RASE of the estimated nonparametric function also becomes smaller and smaller. This reflects that the estimate curves fit better to the corresponding true line as the sample size increases. Moreover, the SCAD penalty method outperforms the LASSO penalty ones in the sense of correct variable selection rate, which significantly reduces the model uncertainty and complexity.

To study the influence of misspecified correlation structure to the proposed approach, we perform variable selection when the working correlation structure is specified to be CS and first-order autoregressive (AR-1), respectively. The result is listed in Table 5. It is known that the QIF estimator is insensitive to misspecification in correlation structure. Table 5 shows that the proposed variable selection procedure gives similar results even when the correlation structure is misspecified. This indicates that our method is robust.

### 4.4. Study 3 (High-Dimensional Setup)

In this example, we discuss how the proposed variable selection procedure can be applied to the “large *n*, diverging *p*/*q*” setup for longitudinal models. We consider the high-dimensional setup of study 2. In this simulation, we take *n* = 300, *m* = 6, *p* = 20 = *O*(*N*^1/4^), *q* = 10 = *O*(*N*^1/4^). The true coefficient vector is *β* = (2,1.5,0.7, 0_17_^*T*^)^*T*^, *α*(*u*) = (*α*_1_(*u*), *α*_2_(*u*), 0_10_^*T*^)^*T*^, where *α*_1_(*u*) and *α*_2_(*u*) are defined in study 2. The other settings are the same with study 2. The results are reported in Table 6. It is easy to see that the proposed variable selection procedure is able to correctly identify the true model and works well in the “large *n*, diverging *p*/*q*” setup.

## 5. Application to Infectious Disease Data

We apply the proposed method to analyze an infectious disease data (indon.data), which has been well analyzed by many authors, such as [16, 23–27]. In this study, a total of 275 preschool children were examined every three months for 18 months. The response is the presence of respiratory infection (1 = yes, 0 = no). The primary interest is in studying the relationship between the risk of respiratory infection and vitamin A deficiency (1 = yes, 0 = no).

In our study, we consider the following GPLVCM model

(30) $$\begin{array}{c} \text{logit}\left\{\mu_{ij}\;|\;X_{ij},t_{ij}\right\}=\overset{6}{\underset{k=1}{\sum }}\;\beta_{i}x_{ij}+\alpha_{0}\left(t_{ij}\right)+z_{ij}\alpha_{1}\left(t_{ij}\right), \end{array}$$

where *t* is age, *X*_1_ is vitamin A deficiency, *X*_2_, *X*_3_ are the seasonal cosine and seasonal sine variables, respectively, which indicate the season when those examinations took place, *X*_4_ is gender (1 = female, 0 = male), *X*_5_ is height, *X*_6_ is stunting status (1 = yes, 0 = no), and *Z*_1_ = *X*_5_^2^ is the square of height. The with-cluster correlation structure is assumed to be exchangeable, i.e., compound symmetric. This structure is also used in [16, 26, 27].

**Figure 1:**
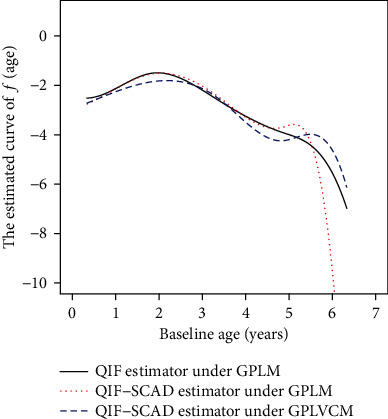
The estimated function on age for the infectious disease data.

We apply the proposed QIF-based group SCAD variable selection procedure to the above model and recognize five nonzero coefficients and one nonzero function *α*_0_(*t*), where *β*_1_ = 0.842, *β*_2_ = −0.685, *β*_3_ = −0.309, *β*_4_ = −0.554, and *β*_6_ = 0.966. The results are generally consistent with those previous studies, but our results show that the height has no significant impact on the infectious rate and can be removed from the model.  Figure 1 reports the curve of baseline age function *α*_0_(*t*) estimated by QIF-based group SCAD that is estimated by QIF and that is estimated by QIF-based SCAD partial penalty to *β* in [16], where the GPLM without the varying coefficient term is used.  Figure 1 implies that the probability of having respiratory infection increases at the very early stage, then decreases steadily, and declines dramatically when the age is over 5.5 years old. This also coincides with previous results [16, 26, 27].

## 6. Conclusion and Discussion

We proposed a QIF-based group SCAD variable selection procedure for the generalized partially linear varying coefficient models with longitudinal data. This procedure can select significant variables in the parametric components and nonparametric components simultaneously. Under mild conditions, the estimators of regression coefficients have oracle property. Simulation studies indicate that the proposed procedure is very effective in selecting significant variables and estimating the regression coefficients.

In this paper, we assume that the dimensions of the covariates *X* and *Z* are fixed. Study 3 in simulations shows that the proposed approach still have desired results when the dimensions *p* and *q* go to infinity as *n*⟶∞. However, when in ultrahigh-dimensional case, the proposed variable selection procedure may not work well anymore. As a future research topic, it is interesting to consider the variable selection for the generalized partially linear varying coefficient models with ultrahigh-dimensional covariates.

## Figures and Tables

**Table 1:** Variable selection for the parametric components under different methods.

	Method	*n* = 150			*n* = 200			*n* = 300		
		GMSE	*C*	IC	GMSE	*C*	IC	GMSE	*C*	IC
*ρ* = 0.75	SCAD	0.0011	15.83	0	0.0006	16.246	0	0.0005	16.746	0
	LASSO	0.0006	14.81	0	0.0005	15.346	0	0.0004	15.574	0

*ρ* = 0.25	SCAD	0.0011	15.75	0	0.0006	16.70	0	0.0004	16.846	0
	LASSO	0.0007	14.82	0	0.0006	14.96	0	0.0005	15.35	0

**Table 2:** RASE of $\hat{\alpha }\left(u\right)$ under different methods.

	Method	*n* = 150	*n* = 200	*n* = 300
*ρ* = 0.75	SCAD	0.1920	0.2051	0.1054
	LASSO	0.0999	0.0840	0.1064

*ρ* = 0.25	SCAD	0.2449	0.2460	0.0694
	LASSO	0.1399	0.1205	0.1033

**Table 3:** Variable selection for the parametric components under different methods.

	Method	*n* = 150			*n* = 200			*n* = 300		
		GMSE	*C*	IC	GMSE	*C*	IC	GMSE	*C*	IC
*ρ* = 0.75	SCAD	0.0048	6.76	0	0.0036	6.846	0	0.0030	6.864	0
	LASSO	0.0039	4.694	0	0.0033	4.766	0	0.0028	5.074	0

*ρ* = 0.25	SCAD	0.0047	6.76	0	0.0035	6.718	0	0.0028	6.846	0
	LASSO	0.0038	4.814	0	0.0035	4.98	0	0.0029	5.048	0

**Table 4:** Variable selection for the nonparametric components under different methods.

	Method	*n* = 150			*n* = 200			*n* = 300		
		GMSE	*C*	IC	GMSE	*C*	IC	GMSE	*C*	IC
*ρ* = 0.75	SCAD	0.1696	4.35	0	0.1221	4.66	0	0.0812	4.83	0
	LASSO	0.1932	4.38	0	0.1540	4.36	0	0.1235	4.57	0

*ρ* = 0.25	SCAD	0.1636	4.42	0	0.1076	4.72	0	0.0344	4.85	0
	LASSO	0.1982	4.40	0	0.1160	4.68	0	0.0398	4.76	0

**Table 5:** Variable selection when the true *R* is CS when *n* = 300.

Working *R*	Method	*β*			*α*(·)		
		GMSE	*C*	IC	RASE	*C*	IC
*ρ* = 0.75							

CS	SCAD	0.0030	6.864	0	0.0812	4.83	0
	LASSO	0.0028	5.074	0	0.1235	4.57	0

AR-1	SCAD	0.0033	6.856	0	0.0935	4.82	0
	LASSO	0.0034	4.924	0	0.1230	4.57	0

*ρ* = 0.25							

CS	SCAD	0.0028	6.846	0	0.0344	4.85	0
	LASSO	0.0029	5.048	0	0.0398	4.76	0

AR-1	SCAD	0.0030	6.846	0	0.0354	4.86	0
	LASSO	0.0031	5.048	0	0.0411	4.75	0

**Table 6:** Variable selection under high-dimensional setup.

	Method	*β*			*α*(·)		
		GMSE	*C*	IC	RASE	*C*	IC
*ρ* = 0.75	SCAD	0.0036	16.664	0	0.1148	9.656	0
	LASSO	0.0033	15.574	0	0.1239	9.546	0

*ρ* = 0.25	SCAD	0.0034	16.846	0	0.1047	9.875	0
	LASSO	0.0039	15.35	0	0.1138	9.802	0

## Data Availability

The data can be downloaded from https://content.sph.harvard.edu/xlin/dat/indon.dat.

## Appendix

### A. Proofs of the Main Results

For convenience and simplicity, let *C* denote a positive constant that may have different values at each appearance throughout this paper and ‖*A*‖ denote the modulus of the largest singular value of matrix or vector *A*.

Let *η*_*ij*_ = *X*_*ij*_^*T*^*β* + *Z*_*ij*_^*T*^ · *I*_*q*_ ⊗ *B*(*U*_*ij*_)^*T*^*γ*, then *μ*_*ij*_ = *h*(*η*_*ij*_). Let *η*_*i*_ = (*η*_*i*1_, ⋯,*η*_*im*_)^*T*^, *μ*_*i*_ = (*μ*_*i*1_, ⋯,*μ*_*im*_)^*T*^, and *θ* = (*β*^*T*^, *γ*^*T*^)^*T*^, *Y*_*i*_ = (*Y*_*i*1_, ⋯,*Y*_*im*_)^*T*^, *X*_*i*_ = (*X*_*i*1_, ⋯,*X*_*im*_)^*T*^.

Similarly, let *W*_*ij*_ = *B*(*U*_*ij*_) ⊗ *I*_*q*_ · *Z*_*ij*_, *P*_*ij*_ = (*X*_*ij*_^*T*^, *W*_*ij*_)^*T*^, and *W*_*i*_ = (*W*_*i*1_, ⋯,*W*_*im*_))^*T*^, *P*_*i*_ = (*P*_*i*1_, ⋯,*P*_*im*_)^*T*^ = (*X*_*i*_, *W*(*U*_*i*_)); then, *η*_*ij*_ = *P*_*ij*_^*T*^*θ*, *η*_*i*_ = *P*_*i*_*θ*, and *∂η*_*ij*_/*∂θ* = *P*_*ij*_, *∂η*_*i*_/*∂θ* = *P*_*i*_^*T*^.

Let *h*′(*t*) = *dh*(*t*)/*dt*, then *∂μ*_*ij*_/*∂θ* = *h*′(*η*_*ij*_)*P*_*ij*_. Let

(A.1) $$\begin{array}{c} H^{\prime }\left(\eta_{i}\right)≜\left(\begin{array}{ccc} h^{\prime }\left(\eta_{i1}\right) & & \\ & \ddots & \\ & & h^{\prime }\left(\eta_{im}\right) \end{array}\right),H^{\prime \prime }\left(\eta_{i}\right)≜\left(\begin{array}{ccc} h^{\prime \prime }\left(\eta_{i1}\right) & & \\ & \ddots & \\ & & h^{\prime \prime }\left(\eta_{i,}\right) \end{array}\right). \end{array}$$

Then,

(A.2) $$\begin{array}{c} {\dot{\mu }}_{i}=\left(\begin{array}{ccc} \frac{\partial \mu_{i1}}{\partial \beta_{1}} & \cdots & \frac{\partial \mu_{i1}}{\partial \gamma_{qL}}\\ \vdots & \cdots & \vdots \\ \frac{\partial \mu_{im}}{\partial \beta_{1}} & \cdots & \frac{\partial \mu_{im}}{\partial \gamma_{qL}} \end{array}\right)=\left(\begin{array}{c} {\left(\frac{\partial \mu_{i1}}{\partial \theta }\right)}^{T}\\ \vdots \\ {\left(\frac{\partial \mu_{im}}{\partial \theta }\right)}^{T} \end{array}\right)=\left(\begin{array}{c} P_{i1}^{T}h^{\prime }\left(\eta_{i1}\right)\\ \vdots \\ P_{im}^{T}h^{\prime }\left(\eta_{im}\right) \end{array}\right)=H^{\prime }\left(\eta_{i}\right)P_{i}. \end{array}$$

Proof of Theorem 1. Let *δ* = *n*^−1/2^, *β* = *β*_0_ + *δD*_1_, *γ* = *γ*_0_ + *δD*_2_, and *D* = (*D*_1_^*T*^, *D*_2_^*T*^)^*T*^. We first show that for any given *ε* > 0, there exists a large constant *C* such that

(A.3) $$\begin{array}{c} P\left\{\underset{\left\|D\right\|=C}{\inf}Q_{n}^{P}\left(\beta ,\gamma \right)>Q_{n}^{P}\left(\beta_{0},\gamma_{0}\right)\right\}\geq 1-\varepsilon . \end{array}$$

Note that *β*_0*l*_ = 0, for all *l* = *P*_1_ + 1, ⋯, *p*, and *γ*_0*k*_ = 0, for all *k* = *q*_1_, ⋯, *q*, together with Assumption (A1) and *p*_*λ*_(0) = 0, we have

(A.4) $$\begin{array}{c} Q_{n}^{p}\left(\theta \right)-Q_{n}^{p}\left(\theta_{0}\right)⩾\left[Q_{n}\left(\theta \right)-Q_{n}\left(\theta_{0}\right)\right]+n\overset{p_{1}}{\underset{l=1}{\sum }}\;\left[p_{\lambda_{2}}\left(\left|\beta_{l}\right|\right)-p_{\lambda_{2}}\left(\left|\beta_{0l}\right|\right)\right]+n\overset{q_{1}}{\underset{k=1}{\sum }}\;\left[p_{\lambda_{1}}\left({\left\|\gamma_{k}\right\|}_{H}\right)-p_{\lambda_{1}}\left({\left\|\gamma_{0k}\right\|}_{H}\right)\right]≜I_{1}+I_{2}+I_{3}. \end{array}$$

By Taylor expansion and Assumption (A4), we have

(A.5) $$\begin{array}{c} I_{2}=n\overset{p_{1}}{\underset{l=1}{\sum }}\;\left[\delta p\prime_{\lambda_{2}}\left(\left|\beta_{0l}\right|\right)\mathrm{sgn}\left(\beta_{0l}\right)\left|D_{1l}\right|+\delta p\prime \prime_{\lambda_{2}}\left(\left|\beta_{0l}\right|\right)\mathrm{sgn}\left(\beta_{0l}\right){\left|D_{1l}\right|}^{2}\left\{1+o\left(1\right)\right\}\right]⩽\sqrt{p_{1}}a_{n}\left\|D\right\|O\left(n^{1/2}\right)+b_{n}{\left\|D\right\|}^{2}O\left(1\right)=\sqrt{p_{1}}\left\|D\right\|O\left(n^{-1/2}\right)+{\left\|D\right\|}^{2}o\left(1\right). \end{array}$$

Invoking the proof of Theorem 2 in Zhang and Xue [16],

(A.6) $$\begin{array}{c} I_{1}=Q_{n}\left(\theta \right)-Q_{n}\left(\theta_{0}\right)=D^{T}{\dot{g}}_{N}^{T}\left(\theta_{0}\right)\Omega_{n}^{-1}\left(\theta_{0}\right){\dot{g}}_{N}\left(\theta_{0}\right)D+{\left\|D\right\|}^{2}o_{p}\left(1\right)+\left\|D\right\|O_{p}\left(1\right). \end{array}$$

By choosing a sufficient large *C*, *I*_1_ dominates *I*_2_. Similarly, *I*_1_ dominates *I*_3_ for a sufficient large *C*. Thus (A.3) holds, i.e., with probability at least 1 − *ε*, there exists a local minimizer $\hat{\theta }$ that satisfies $\left\|\hat{\theta }-\theta_{0}\right\|=O_{p}\left(\delta \right)$. Therefore, $\left\|\hat{\gamma }-\gamma_{0}\right\|=O_{p}\left(n^{-1/2}\right)$ and $\left\|\hat{\beta }-\beta_{0}\right\|=O_{p}\left(n^{-1/2}\right)$. Let *R*_*k*_(*u*) = *α*_*k*_(*u*) − *B*(*U*)^*T*^*γ*_*k*_ and *γ*_*ok*_ denote the spline coefficient vector from the spline approximation to *α*_*k*_(·). From Assumptions (A7) and (A8) and Theorem 12.7 in [18], we get that ‖*R*_*k*_(*u*)‖ = *O*(*K*^−*r*^). Therefore,

(A.7) $$\begin{array}{c} {\left\|{\overset{\wedge }{\alpha }}_{k}\left(u\right)-\alpha_{0k}\left(u\right)\right\|}^{2}=\int_{0}^{1}\;{\left\{{\overset{\wedge }{\alpha }}_{k}\left(u\right)-\alpha_{0k}\left(u\right)\right\}}^{2}\;du=\int_{0}^{1}\;{\left\{B{\left(u\right)}^{T}{\overset{\wedge }{\gamma }}_{k}-B{\left(u\right)}^{T}\gamma_{0k}+R_{k}\left(u\right)\right\}}^{2}\;du\leq 2\int_{0}^{1}\;{\left\{B{\left(u\right)}^{T}{\overset{\wedge }{\gamma }}_{k}-B{\left(u\right)}^{T}\gamma_{0k}\right\}}^{2}\;du+2\int_{0}^{1}\;R_{k}{\left(u\right)}^{2}\;du=2{\left({\overset{\wedge }{\gamma }}_{k}-\gamma_{0k}\right)}^{T}\int_{0}^{1}\;B\left(u\right)B^{T}\left(u\right)\;du\;\left({\hat{\gamma }}_{k}-\gamma_{0k}\right)+2\int_{0}^{1}\;R_{k}{\left(u\right)}^{2}\;du=O_{p}\left(n^{-2r/\left(2r+1\right)}\right). \end{array}$$

Thus, we complete the proof of Theorem 1.

Proof of Theorem 2. According to Theorem 2, in order to prove the first part of Theorem 2, we need only to prove that, for any *γ* satisfying ‖*γ* − *γ*_0_‖ = *O*_*p*_(*n*^−1/2^) and for any *β*_*l*_ satisfying ‖*β*_*l*_ − *β*_0*l*_‖ = *O*_*p*_(*n*^−1/2^), *l* = 1, ⋯, *p*_1_, there exists a certain *ϵ* = *Cn*^−1/2^ that satisfies, as *n*⟶∞, with probability tending to 1:

(A.8) $$\begin{array}{c} \frac{\partial Q_{n}^{p}\left(\beta ,\gamma \right)}{\partial \beta_{l}}>0,\text{for}\;0<\beta_{l}<\epsilon ,l=p_{1}+1,\cdots ,p, \end{array}$$

(A.9) $$\begin{array}{c} \frac{\partial Q_{n}^{p}\left(\beta ,\gamma \right)}{\partial \beta_{l}}<0,\text{for}\;-\epsilon <\beta_{l}<0,l=p_{1}+1,\cdots ,p. \end{array}$$

These imply that the PQIF 𝒬_*n*_^*p*^(*β*, *γ*) reaches its minimum at *β*_*l*_ = 0, *l* = *p*_1_ + 1, ⋯, *p*. Following Lemmas 3 and 4 of [16], we have

(A.10) $$\begin{array}{c} \frac{\partial Q_{n}^{p}\left(\beta ,\gamma \right)}{\partial \beta_{l}}=\frac{\partial g_{n}^{T}\left(\beta ,\gamma \right)}{\partial \beta_{l}}\Omega_{n}^{-1}\left(\beta ,\gamma \right)g_{n}\left(\beta ,\gamma \right)+O_{p}\left(1\right)+np\prime_{\lambda_{2}\left(\;|\;\beta_{l}\;|\;\right)}\;\mathrm{sgn}\;\left(\beta_{l}\right)=-2\overset{n}{\underset{i=1}{\sum }}\;{\left(\begin{array}{c} {\dot{\mu }}_{i}^{T}A_{i}^{-1/2}M_{1}A_{i}^{-1/2}\frac{\partial \mu_{i}}{\partial \beta_{l}}\\ \vdots \\ {\dot{\mu }}_{i}^{T}A_{i}^{-1/2}M_{s}A_{i}^{-1/2}\frac{\partial \mu_{i}}{\partial \beta_{l}} \end{array}\right)}^{T}\Omega_{n}^{-1}\left(\beta ,\gamma \right)g_{n}\left(\beta ,\gamma \right)+np\prime_{\lambda_{2}\left(\;|\;\beta_{l}\;|\;\right)}\;\mathrm{sgn}\;\left(\beta_{l}\right)+O_{p}\left(1\right)=n^{1/2}\left[n^{1/2}\lambda_{2}\left\{\lambda_{2}^{-1}p\prime_{\lambda_{2}}\left(\;|\;\beta_{l}\;|\;\right)\;\mathrm{sgn}\;\left(\beta_{l}\right)\right\}+O_{p}\left(1\right)\right]. \end{array}$$

According to (8), the expression of the derivative of SCAD-penalized function, it is easy to see that lim_*n*→∞_liminf_*β*_*l*_→0_*λ*_2_^−1^*p*′_*λ*_2__(∣*β*_*l*_∣) = 1. Together with Assumption (A10), *λ*_2_*n*^1/2^ > *λ*_min_*n*^1/2^⟶∞, it is clear that the sign of (A.10) is decided by that of *β*_*l*_. This implies (A.8) and (A.9) hold. Thus, we complete the proof of the first part. Similarly, we can prove that with probability tending to 1, ${\hat{\gamma }}_{k}=0,k=q_{1}+1,\cdots ,q$. Note that ‖*B*(*u*)‖ = *O*(1) and ${\hat{\alpha }}_{k}\left(u\right)=B^{T}\left(u\right){\hat{\gamma }}_{k}$; the second part of Theorem 2 is proved. Thus, we complete the proof of Theorem 2.

Proof of Theorem 3. Let *θ*^∗^ = (*β*^∗*T*^, *γ*^∗*T*^)^*T*^ and let *P*_*i*_^∗^ = (*X*_*i*_^∗*T*^, *W*_*i*_^∗*T*^)^*T*^, *i* = 1, ⋯, *n* denote the covariates corresponding to *θ*^∗^. Denote ${\dot{𝒬}}_{1n}\left(\beta ,\gamma \right)$ and ${\dot{𝒬}}_{2n}\left(\beta ,\gamma \right)$ to be the first derivatives of the PQIF 𝒬_*n*_^*p*^ with respect to *β* and *γ*, respectively, i.e.,

(A.11) $$\begin{array}{c} {\dot{Q}}_{1n}\left(\beta ,\gamma \right)=\frac{\partial Q_{n}^{p}\left(\beta ,\gamma \right)}{\partial \beta },\\ {\dot{Q}}_{2n}\left(\beta ,\gamma \right)=\frac{\partial Q_{n}^{p}\left(\beta ,\gamma \right)}{\partial \gamma }. \end{array}$$

By Theorems 1 and 2, ${\left({\overset{\wedge }{\beta }}^{*T},0^{T}\right)}^{T}$ and ${\left({\overset{\wedge }{\gamma }}^{*T},0^{T}\right)}^{T}$ satisfies that

(A.12) $$\begin{array}{c} {\dot{Q}}_{1n}\left({\left({\overset{\wedge }{\beta }}^{*T},0^{T}\right)}^{T},{\left({\overset{\wedge }{\gamma }}^{*T},0^{T}\right)}^{T}\right)=0^{T},\\ {\dot{Q}}_{2n}\left({\left({\overset{\wedge }{\beta }}^{*T},0^{T}\right)}^{T},{\left({\overset{\wedge }{\gamma }}^{*T},0^{T}\right)}^{T}\right)=0^{T}. \end{array}$$

By the Taylor expansion, we have

(A.13) $$\begin{array}{c} Q_{1n}{\left|{}_{\left({\left({\overset{\wedge }{\beta }}^{*T},0^{T}\right)}^{T},{\left({\overset{\wedge }{\gamma }}^{*T},0^{T}\right)}^{T}\right)}=Q_{1n}\right|}_{\left(\left(\beta_{0}^{*T},0^{T}\right){\left(\beta_{0}^{*T},0^{T}\right)}^{T},{\left(\gamma_{0}^{*T},0^{T}\right)}^{T}\right)}+\frac{\partial Q_{1n}}{\partial \beta }{\left|{}_{\theta =\tilde{\theta }}\left\{{\left({\overset{\wedge }{\beta }}^{*T},0^{T}\right)}^{T}-{\left(\beta_{0}^{*T},0^{T}\right)}^{T}\right\}+\frac{\partial Q_{1n}}{\partial \gamma }\right|}_{\theta =\tilde{\theta }}\left\{{\left({\overset{\wedge }{\gamma }}^{*T},0^{T}\right)}^{T}-{\left(\gamma_{0}^{*T},0^{T}\right)}^{T}\right\}+\overset{p_{1}}{\underset{i=1}{\sum }}\;np\prime_{\lambda_{2}}\left({\hat{\beta }}_{l}\right)\mathrm{sgn}\left({\hat{\beta }}_{l}\right), \end{array}$$

where $\tilde{\theta }$ is between ((*β*_0_^∗*T*^, 0^*T*^)^*T*^, (*γ*_0_^∗*T*^, 0^*T*^)^*T*^) and $\left({\left({\overset{\wedge }{\beta }}^{*T},0^{T}\right)}^{T},{\left({\overset{\wedge }{\gamma }}^{*T},0^{T}\right)}^{T}\right)$. Apply the Taylor expansion to $p\prime_{\lambda_{2}}\left(|{\hat{\beta }}_{l}|\right)$, we obtain

(A.14) $$\begin{array}{c} p\prime_{\lambda_{2}}\left(\left|{\hat{\beta }}_{l}\right|\right)=p\prime_{\lambda_{2}}\left(\left|\beta_{0l}\right|\right)+\left\{p\prime \prime_{\lambda_{2l}}\left(\left|\beta_{0l}\right|\right.+o_{p}\left(1\right)\right\}\left({\hat{\beta }}_{l}-\beta_{0l}\right). \end{array}$$

By Assumption (A10), *p*′′_*λ*_2__(∣*β*_0*l*_∣) = *o*_*p*_(1). Note that *p*′_*λ*_2*l*__(∣*β*_0*l*_∣) = 0 as *λ*_max_⟶0; therefore, by Lemma 4 of [16] and through some calculation, we have

(A.15) $$\begin{array}{c} {\left.\frac{1}{n}Q_{1n}\right|}_{\left({\left(\beta_{0}^{*T},0^{T}\right)}^{T},{\left(\gamma_{0}^{*T},0^{T}\right)}^{T}\right)}=-\frac{2}{n^{2}}\overset{n}{\underset{i=1}{\sum }}\;\overset{n}{\underset{j=1}{\sum }}\;\overset{s}{\underset{k=1}{\sum }}\;\overset{s}{\underset{l=1}{\sum }}\;\left\{X_{i}^{*T}H^{\prime }\left(\eta_{i}\right)A_{i}^{-1/2}M_{k}A_{i}^{-1/2}H^{\prime }\left(\eta_{i}\right)P_{i}^{*}\cdot \Omega_{kl}^{-1}P_{j}^{*T}H^{\prime }\left(\eta_{j}\right)A_{j}^{-1/2}M_{l}A_{j}^{-1/2}\left(Y_{j}-\mu_{0j}\right)\right\}+o_{p}\left(n^{-1/2}\right)=-\frac{2}{n^{2}}\overset{n}{\underset{i=1}{\sum }}\;\overset{n}{\underset{j=1}{\sum }}\;X_{i}^{*T}H^{\prime }\left(\eta_{i}\right)\tau_{ij}\left(Y_{j}-\mu_{0j}\right)+o_{p}\left(n^{-1/2}\right)=-\frac{2}{n^{2}}\overset{n}{\underset{i=1}{\sum }}\;\overset{n}{\underset{j=1}{\sum }}\;{\tilde{X}}_{i}^{T}\tau_{ij}\left(\tilde{R}\left(U_{j}\right)+\varepsilon_{j}\right)+o_{p}\left(n^{-1/2}\right), \end{array}$$

where ${\tilde{X}}_{i}=H^{\prime }\left(\eta_{i}\right)X_{i}^{*},\tilde{R}\left(U_{i}\right)=H^{\prime }\left(\eta_{i}\right)R\left(U_{i}\right),\Omega_{kl}^{-1}$ is the (*l*, *k*) block of *Ω*^−1^ and

(A.16) $$\begin{array}{c} \tau_{ij}=\overset{s}{\underset{k=1}{\sum }}\;\overset{s}{\underset{l=1}{\sum }}\;A_{i}^{-1/2}M_{k}A_{i}^{-1/2}H^{\prime }\left(\eta_{i}\right)P_{i}^{*}\Omega_{kl}^{-1}P_{j}^{*T}H^{\prime }\left(\eta_{j}\right)A_{j}^{-1/2}M_{l}A_{j}^{-1/2}. \end{array}$$

Similarly, we have

(A.17) $$\begin{array}{c} \begin{array}{cc} {\left.\frac{1}{n}\frac{\partial Q_{1n}}{\partial \beta }\right|}_{\theta =\overset{\sim }{\theta }} & =-\frac{2}{n^{2}}\overset{n}{\underset{i=1}{\sum }}\;\overset{n}{\underset{j=1}{\sum }}\;{\tilde{X}}_{i}^{T}\tau_{ij}{\tilde{X}}_{j}+o_{p}\left(n^{-1/2}\right), \end{array}\\ {\left.\frac{1}{n}\frac{\partial Q_{1n}}{\partial \gamma }\right|}_{\theta =\tilde{\theta }}=-\frac{2}{n^{2}}\overset{n}{\underset{i=1}{\sum }}\;\overset{n}{\underset{j=1}{\sum }}\;{\tilde{X}}_{i}^{T}\tau_{ij}\tilde{W}\left(U_{j}\right)+o_{p}\left(n^{-1/2}\right), \end{array}$$

where $\tilde{W}\left(U_{j}\right)=H^{\prime }\left(\eta_{j}\right)W^{*}\left(U_{j}\right),W^{*}\left(U_{j}\right)={\left(W_{j1}^{*},\cdots ,W_{jm}^{*}\right)}^{T},W_{ij}^{*}=B\left(U_{ij}\right)⊗I_{q}\cdot Z_{ij}^{*}$. Hence,

(A.18) $$\begin{array}{c} {\left.\frac{1}{n}Q_{1n}\right|}_{\left({\left(\beta_{0}^{*T},0^{T}0\right)}^{T},{\left(\gamma_{0}^{*T},0^{T}0\right)}^{T}\right)}=-\frac{2}{n^{2}}\overset{n}{\underset{i=1}{\sum }}\;\overset{n}{\underset{j=1}{\sum }}\;{\tilde{X}}_{i}^{T}\tau_{ij}\left\{{\tilde{X}}_{j}\left(\beta_{0}^{*}-{\overset{\wedge }{\beta }}^{*}\right)+\tilde{W}\left(U_{j}\right)\cdot \left(\gamma_{0}^{*}-{\overset{\wedge }{\gamma }}^{*}\right)+\tilde{R}\left(U_{j}\right)+\varepsilon_{j}\right\}+o_{p}\left({\overset{\wedge }{\beta }}^{*}-\beta_{0}^{*}\right),\\ {\left.\frac{1}{n}Q_{2n}\right|}_{\left({\left(\beta_{0}^{*T},0^{T}0\right)}^{T},{\left(\gamma_{0}^{*T},0^{T}0\right)}^{T}\right)}=-\frac{2}{n^{2}}\overset{n}{\underset{i=1}{\sum }}\;\overset{n}{\underset{j=1}{\sum }}\;\tilde{W}{\left(U_{i}\right)}^{T}\tau_{ij}\left\{{\tilde{X}}_{j}\left(\beta_{0}^{*}-{\overset{\wedge }{\beta }}^{*}\right)+\tilde{W}\left(U_{j}\right)\cdot \left(\gamma_{0}^{*}-{\overset{\wedge }{\gamma }}^{*}\right)+\tilde{R}\left(U_{j}\right)+\varepsilon_{j}\right\}+o_{p}\left({\overset{\wedge }{\gamma }}^{*}-\gamma_{0}^{*}\right). \end{array}$$

Following the proof of Theorem 2 in [16], we prove (16). Thus, we complete the proof of Theorem 3.

## Supplementary Materials

Supplementary Materials The R code presented in Word format for the real data analysis is included in the supplementary file.
