# Supplementary material for: Penalized Quadratic Inference Function-Based Variable Selection for Generalized Partially Linear Varying Coefficient Models with Longitudinal Data
Source: Comput Math Methods Med. 2020 Oct 5;2020:3505306. doi: 10.1155/2020/3505306 (PMC7556090; doi:10.1155/2020/3505306)
Supplement: Supplementary Materials — The R code presented in Word format for the real data analysis is included in the supplementary file. [file 3505306.f1.docx]

Five R scripts are included.

RealDataAllData.R : the program of QIF-estimator for the real data. The result is saved in RealDateAllData.txt.

realdata-scad.R : the program of QIF-SCAD under GPLM for the real data. The result is saved in RealDataScad.txt.

real-scad.R: Main program of QIF-SCAD under GPLVM for the real data, while the degree of fredom of the model is defined as Xue, Qu and Zhou [9]; The result is saved in RealDataScad-xue.txt

real-scad-tian.R : Main program of QIF-SCAD under GPLVM for the real data, while the degree of fredom of the model is defined as Tian,Xue and Liu. [15]; The result is saved in RealDateScad-tian.txt.

realgains.R : all sub-program needed for real-scad.r and real-scad-tian.r.

funcompare.R : the program to plot the estimated curve in 3 setup: GPLM with no penalty, GPLM with SCAD penalty and GPLVM with SCAD penalty.

**RealDataAllData.R: the program of QIF-estimator for the real data. The result is saved in RealDateAllData.txt.**

###################################################################

##### 纵向数据广义部分线性模型

##### 实例分析--呼吸道感染数据

##### 数据来源--https://www.hsph.harvard.edu/xlin/data.html

###################################################################

#### id --> 个体的编号,个体按编号排序,不同的ID对应不同的个体

#### np --> 所研究的数据集包含的变量个数

#### obs --> 向量,每个分量表示相应个体的观测次数

#### nobs--> 个体的最大观测次数.

#### delobs--> 观测次数少于nobs的的个体的ID对应的位置

#### temp --> 数据按ID分组

#### nsub --> 数据所包含的个体总数

#### *************************************************************

#### loc1 --> 第i个个体第一次观测所在的行号

#### loc2 --> 第i个个体最后一次观测所在的行号

#### *************************************************************

#### indon数据一共14列,从左至右的14个指标分别是--

#### v1. 不同个体的识别ID;

#### v2. 呼吸道是否感染: 1-感染 vs 0-未感染;

#### v3. 截距系数,取1;

#### v4. 年龄: 真实年龄(月数)-36;

#### v5. 眼睛是否干涩: 1-是 vs 0-否;

#### v6. 季节的正弦: sin(visit);

#### v7. 季节的余弦: cos(visit);

#### v8. 性别: 1-女性 vs 0-男性;

#### v9. 身高;

#### v10. 是否头晕: 1-是 vs 0-否;

#### v11. 第k次观测: k=1-6;

#### v12. baseline age;

#### v13. 季节指标: 1-春,2-夏,3-秋,4-冬;

#### v14. k*k

#### *************************************************************

#### 建模--响应变量为v2,协变量-v5+v6+v7+v8+v9+v10,非参协变量-v12

#### *************************************************************

#### split(y,x) -->将数据集y按x分组

###################################################################

rm(list=ls())

library(MASS)

library(splines)

dat<-read.table("indon.dat",header=FALSE)

dat<-as.matrix(dat)

np<-ncol(dat)

id<-dat[,1]

obs<-lapply(split(id,id),"length")

obs<-as.numeric(obs)

nobs<-max(obs)

temp<-split(dat,id)

nsub<-length(temp)

time_start=Sys.time()

################################################################

####### 计算初值 ####

##### ************************************************* ####

##### 在组内独立假定下求初值,这是因为根据GEE的理论结果, ##

##### 这样得到的初值仍是根方相合的. ##

##### 在组内独立假定下,直接用广义线性模型的估计方法 ##

##### 就可以得到相关参数的估计. ##

##### ************************************************** ##

##### bu-->非参函数在各点的的样条基构成的矩阵,注意 ##

##### 所用的bs命令得到的样条基是不含截距项的. ##

##### tet.ini-->参数beta和样条基系数gamma的初值. ##

##### 注意tet.ini中含截距项. ##

##############################################################

y<- dat[,2]

x<- dat[,5:10]

u<- dat[,12]

bu<- bs(u,df=6)

tet.ini<- glm(formula=y~x+bu,family=binomial)$coefficients

tet.ini<- as.vector(tet.ini)

#################################################################

#### n--> 样本量 ##

#### m--> 样本重复观测次数 ##

#### va--> 样本标准差 ##

#### L--> B样条基的阶数,取L=6的3次B样条 ##

#### np--> 参数的个数+1(截距项的维数) ##

#### npe--> 对非参函数作样条逼近后,待估参数的个数 ##

#### nb--> 基矩阵中的矩阵的个数,对CS结构,nb=2 ##

#### ne--> 估计方程个数=npxnb ##

#### M1--> CS结构对应的基矩阵之一,为单位阵 ##

#### M2--> CS结构对应的基矩阵之二,对角元为零, ##

#### 非对角元均为1. ##

#### ***************************************************** ##

#### 以下为预定义的初值 ##

#### ***************************************************** ##

#### PB --> 伪设计阵,由截距、X和样条基构成 ##

#### gi --> 广义score向量, dx1维 ##

#### dgi--> gi的一阶偏导数矩阵, dxp维 ##

#### dQn1-> 估计函数Qn的一阶偏导数的第一部分, p维 ##

#### tet.hat--> 全部参数的估计值 ##

#### it_max--> 求根时的迭代次数上限 ##

#### tolerance--> 判断所得估计是否收敛的阈值 ##

#################################################################

#################################################################

n<-nsub

N=length(id)

L=6

np<-7

npe<- np+L

nb=2

ne<-npe*nb

gi<-rep(0,ne)

dgi<-matrix(0,ne,npe)

dQn1<-rep(0,npe)

loc2<-0

it_max<-500

tolerance<-1e-7

fails=0

##############################################################

########## 子程序--迭代公式中的 d2Q^{-1}*dQ ########

############################################################

#### 迭代求解中需要的一系列中间结果 ########

#### ********************************************** ########

#### ne--->广义Score向量的维数(参数个数x基矩阵个数) ####

#### gi,gn,omega-->定义见QIF方法中的定义,此处略 ####

#### gi--> d维列向量 ####

#### gn--> d维列向量 ####

#### omega--> d行xd列的矩阵 ####

#### dgi-->gi关于thtea的一阶偏导数, d行xp列的矩阵 ####

#### dgn-->gn关于thtea的一阶偏导数, d行xp列的矩阵 ####

#### domega_i-->omega关于theta_i的一阶偏导数矩阵 ####

#### ********************************************** ########

#### 因为设计阵X中没有全1列,所以在迭代求解时, ########

#### 需要给样条基矩阵加一个全1列以容纳截距项. ########

#### ********************************************** ########

#### loc1,loc2-->第i个个体m次观测的所在的位置 ####

##############################################################

renew<-function(tet) {

intercept<- tet[1]

bta<- tet[2:np]

gama<- tet[-(1:np)]

eta<- intercept+x%*%bta+bu%*%gama

mu<- 1/(1+exp(-eta))

dh<-mu*(1-mu)

res<- y-mu

omega<- 0

gn<- 0

dgn<- 0

temp<- rep(0,ne)

for (i in 1:n) {

m <-obs[i]

M1<- diag(m)

M2<- 1*(row(M1)!=col(M1))

PB<-matrix(0,m,npe)

loc1<-loc2+1

loc2<-loc2+obs[i]

PB[,1]<- rep(1,m)

PB[,2:np]<- x[loc1:loc2,]

PB[,(np+1):npe]<- bu[loc1:loc2,]

dmu<- if (loc1==loc2) dh[loc1]*PB else diag(dh[loc1:loc2])%*%PB

gi[1:npe]<- t(PB)%*%M1%*%res[loc1:loc2]

gi[(npe+1):ne]<- t(PB)%*%M2%*%res[loc1:loc2]

omega<-omega+gi%*%t(gi)

gn<-gn+gi

dgi[1:npe,]<- t(PB)%*%M1%*%dmu

dgi[(npe+1):ne,]<- t(PB)%*%M2%*%dmu

dgn<-dgn+dgi

}

omega<-omega/N

omega.inv<-ginv(omega)

gn<-gn/N

dgn<- -dgn/N

dQn<-2*t(dgn)%*%omega.inv%*%gn

ddQn<-2*t(dgn)%*%omega.inv%*%dgn

ddQn.inv<-ginv(ddQn)

tet.new<-ddQn.inv%*%dQn

return(tet.new)

}

################################################################

###### 牛顿迭代求解 ##########

#### ************************************************** ####

#### index-->取0表示迭代发散,未得到解. ####

#### k--> 迭代次数,初值为0 ####

#### distance--> 前后两次估计值的差 ####

#### norm--> 前后两次估计值的差的二范数 ####

################################################################

index=0

k=1

tet<-tet.ini

while (k<=it_max){

tet1<-tet

tet<-tet-renew(tet)

dif<-tet-tet1

norm<-sqrt(t(dif)%*%(dif))

if (norm<tolerance) { index<-1

tet.hat<-tet

break}

k<-k+1

}

if (index==0) print ("求解失败")

intercept.hat<-tet.hat[1]

bta.hat<-tet.hat[2:np]

gama.hat<-tet.hat[(np+1):npe]

names(bta.hat)<-c("Vitamin A","Scos","Ssin","Sex","Height","Stunting")

################################################################

###### 计算标准误 ######

################################################################

eta.hat<- tet[1]+x%*%tet[2:np]+bu%*%tet[-(1:np)]

mu.hat<- 1/(1+exp(-eta.hat))

dh<-mu.hat*(1-mu.hat)

res<- y-mu.hat

loc2<-0

nx<-np-1

omega<- 0

gn<- 0

dgX<- 0

dgB<-0

dgx<-matrix(0,2*npe,nx)

dgb<-matrix(0,2*npe,L)

temp<- rep(0,ne)

for (i in 1:n) {

m <-obs[i]

loc1<-loc2+1

loc2<-loc2+obs[i]

temp<-sqrt(dh[loc1:loc2])

M1<-diag(m)

M2<- 1*(row(M1)!=col(M1))

M1<- if (loc1==loc2) diag(m)/(dh[loc1]) else diag(1/dh[loc1:loc2])

M2<- if (loc1==loc2) M2/(dh[loc1]) else diag(temp)%*%M2%*%diag(temp)

PB<-matrix(0,m,npe)

xi<-x[loc1:loc2,]

BU<-bu[loc1:loc2,]

PB[,1]<- rep(1,m)

PB[,2:np]<- x[loc1:loc2,]

PB[,(np+1):npe]<- bu[loc1:loc2,]

dmu<- if (loc1==loc2) dh[loc1]*PB else diag(dh[loc1:loc2])%*%PB

xi <- if (loc1==loc2) t(as.matrix(dh[loc1]*xi)) else diag(dh[loc1:loc2])%*%x[loc1:loc2,]

BU <- if (loc1==loc2) t(as.matrix(dh[loc1]*BU)) else diag(dh[loc1:loc2])%*%bu[loc1:loc2,]

gi[1:npe]<- t(dmu)%*%M1%*%res[loc1:loc2]

gi[(npe+1):ne]<- t(dmu)%*%M2%*%res[loc1:loc2]

omega<-omega+gi%*%t(gi)

gn<-gn+gi

dgx[1:npe,]<- t(PB)%*%M1%*%xi

dgx[(npe+1):(2*npe),]<- t(PB)%*%M2%*%xi

dgX<-dgX+dgx

dgb[1:npe,]<-t(PB)%*%M1%*%BU

dgb[(npe+1):(2*npe),]<-t(PB)%*%M2%*%BU

dgB<-dgB+dgb

}

omega<-omega/N

omega.inv<-ginv(omega)

gn<-gn/N

dgX<- -dgX/N

dgB<- -dgB/N

PAi<-t(dgX)%*%omega.inv%*%dgX

PSi<-t(dgX)%*%omega.inv%*%dgB

PHi<-t(dgB)%*%omega.inv%*%dgB

PHi.inv<-ginv(PHi)

Gama<-PAi+PSi%*%PHi.inv%*%t(PSi)

Deta1<-t(dgX)%*%omega.inv%*%gn

Deta2<-PSi%*%PHi.inv%*%t(dgB)%*%omega.inv%*%gn

Deta<- Deta1+ Deta2

Deta<- Deta%*%t(Deta)

Gama.inv<-ginv(Gama)

se<-diag(Gama.inv%*%Deta%*%Gama.inv)

se<-sqrt(N*se)

names(se)<-c("Va","Scos","Ssin","sex","Height","stunting")

################################################################

###### 输 出 结 果 ######

###### *********************************************** ######

###### 程序耗时 5.232 Minutes in Thinkpad T530 ######

################################################################

failtimes<-as.data.frame(fails)

u<-runif(300,-32,40)

u<-sort(u)

buu<-bs(u,df=6)

yhat<-intercept.hat+buu%*%gama.hat

xt<-u/12+3

plot(xt,yhat,type="l",col=1,xlim=c(0,7),ylim=c(-8,0),xlab="Baseline age (years)",lwd=2,ylab="f(age)")

text(3.63,-2.3,"f(age)")

list(bta.hat=bta.hat)

list(standard_error=se)

time_end=Sys.time()

time_end-time_start

write(tet.hat,file="RealDataAllData.txt")

**realdata-scad.R: the program of QIF-SCAD under GPLM for the real data. The result is saved in RealDataScad.txt.**

###################################################################

##### 纵向数据广义部分线性模型的变量选择

##### 实例分析--呼吸道感染数据

##### 数据来源--https://www.hsph.harvard.edu/xlin/data.html

###################################################################

rm(list=ls())

##############################################################

########## QIF-SCAD中对theta的更新 ########

############################################################

#### 迭代求解中需要的一系列中间结果 ########

#### ********************************************** ########

#### ne--->广义Score向量的维数(参数个数x基矩阵个数) ####

#### gi,gn,omega-->定义见QIF方法中的定义,此处略 ####

#### gi--> d维列向量 ####

#### gn--> d维列向量 ####

#### omega--> d行xd列的矩阵 ####

#### dgi-->gi关于thtea的一阶偏导数, d行xp列的矩阵 ####

#### dgn-->gn关于thtea的一阶偏导数, d行xp列的矩阵 ####

#### domega_i-->omega关于theta_i的一阶偏导数矩阵 ####

#### ********************************************** ########

#### 因为设计阵X中没有全1列,所以在迭代求解时, ########

#### 需要给样条基矩阵加一个全1列以容纳截距项. ########

#### ********************************************** ########

#### loc1,loc2-->第i个个体m次观测的所在的位置 ####

##############################################################

renew<-function(tet) {

np<-length(tet)

ne<-np*nbm

p1<-np-L

intercept<-tet[1]

bta<- if (p1>1) tet[2:p1] else 0

gama<- tet[-(1:p1)]

eta<-if (p1>2) intercept+X1%*%bta+bu%*%gama else intercept+X1*bta+bu%*%gama

mu<- 1/(1+exp(-eta))

dh<-mu*(1-mu)

res<- y-mu

omega<- 0

gn<- 0

dgn<- 0

loc2<-0

temp<- rep(0,ne)

gi<-rep(0,ne)

dgi<-matrix(0,ne,np)

dQn1<-rep(0,np)

for (i in 1:nsub) {

m <-obs[i]

M1<- diag(m)

M2<- 1*(row(M1)!=col(M1))

PB<-matrix(0,m,np)

loc1<-loc2+1

loc2<-loc2+m

PB[,1]<-rep(1,m)

if (p1!=1) {PB[,2:p1]<-if (p1>2) X1[loc1:loc2,] else X1[loc1:loc2]}

PB[,(p1+1):np]<- bu[loc1:loc2,]

dmu<- if (loc1==loc2) dh[loc1]*PB else diag(dh[loc1:loc2])%*%PB

gi[1:np]<- t(PB)%*%M1%*%res[loc1:loc2,]

gi[(np+1):ne]<- t(PB)%*%M2%*%res[loc1:loc2,]

omega<-omega+gi%*%t(gi)

gn<-gn+gi

dgi[1:np,]<- t(PB)%*%M1%*%dmu

dgi[(np+1):ne,]<- t(PB)%*%M2%*%dmu

dgn<-dgn+dgi

}

omega<-omega/nsub

omega.inv<-ginv(omega)

gn<-gn/nsub

dgn<- -dgn/nsub

dQn<-2*t(dgn)%*%omega.inv%*%gn

ddQn<-2*t(dgn)%*%omega.inv%*%dgn

ddQn.inv<-ginv(ddQn+nsub*Sig_lamda(tet,lam))

tet.new<-ddQn.inv%*%(dQn+nsub*Sig_lamda(tet,lam)%*%tet)

return(tet.new)

}

#################################################################

### SCAD惩罚目标函数Qn_p

### Qn(tet) QIF估计的目标函数

### p_scad(tet,lam,a=3.7) SCAD惩罚函数

#################################################################

Qn<-function(tet) {

np<-length(tet)

ne<-np*nbm

p1<-np-L

intercept<-tet[1]

bta<- tet[2:p1]

gama<- tet[-(1:p1)]

eta<- intercept+x%*%bta+bu%*%gama

mu<- 1/(1+exp(-eta))

dh<-mu*(1-mu)

res<- y-mu

omega<- 0

gn<- 0

dgn<- 0

loc2<-0

temp<- rep(0,ne)

gi<-rep(0,ne)

dgi<-matrix(0,ne,np)

dQn1<-rep(0,np)

for (i in 1:nsub) {

m <-obs[i]

M1<- diag(m)

M2<- 1*(row(M1)!=col(M1))

PB<-matrix(0,m,np)

loc1<-loc2+1

loc2<-loc2+m

PB[,1]<-rep(1,m)

PB[,2:p1]<- x[loc1:loc2,]

PB[,(p1+1):np]<- bu[loc1:loc2,]

dmu<- if (loc1==loc2) dh[loc1]*PB else diag(dh[loc1:loc2])%*%PB

gi[1:np]<- t(PB)%*%M1%*%res[loc1:loc2,]

gi[(np+1):ne]<- t(PB)%*%M2%*%res[loc1:loc2,]

omega<-omega+gi%*%t(gi)

gn<-gn+gi

}

omega<-omega/nsub

omega.inv<-ginv(omega)

gn<-gn/nsub

value<-nsub*t(gn)%*%omega.inv%*%gn

return(value)

}

p_scad<-function(tet,lam,a=3.7){

temp<-sapply(tet, function(x){

if(abs(x)< lam) return (lam*abs(x))

else if (abs(x)>=lam & abs(x)<=a*lam) return ((x^2+lam^2 -2*a*lam*abs(x))/(2-2*a))

else return ((a+1)^2*lam^2/2)

}

)

temp<-sum(temp)

return(temp)

}

#################################################################

### SCAD惩罚函数的导数value及迭代式中的惩罚矩阵Sig_lam

#################################################################

Sig_lamda<-function(tet,lamda,a=3.7){

len.tet<-length(tet)

if (len.tet>7) {

fun<-tet[2:(len.tet-L)]

tet<-abs(fun)

temp<-which(tet!=0)

fun[temp]<-1/tet[temp]

value<-0*tet

value[which(tet<lamda)]<-lamda

index<-which(tet>lamda & tet<a*lamda)

value[index]<-(a*lamda-tet[index])/(a-1)

sig_lam<-diag(c(0,value*fun,rep(0,L)))}

else sig_lam<-diag(rep(0,7))

sig_lam

}

#########################################################################

#### id --> 个体的编号,个体按编号排序,不同的ID对应不同的个体

#### np --> 所研究的数据集包含的变量个数

#### obs --> 向量,每个分量表示相应个体的观测次数

#### nobs--> 个体的最大观测次数.

#### delobs--> 观测次数少于nobs的的个体的ID对应的位置

#### temp --> 数据按ID分组

#### nsub --> 数据所包含的个体总数

#### *************************************************************

#### loc1 --> 第i个个体第一次观测所在的行号

#### loc2 --> 第i个个体最后一次观测所在的行号

#### *************************************************************

#### indon数据一共14列,从左至右的14个指标分别是--

#### v1. 不同个体的识别ID;

#### v2. 呼吸道是否感染: 1-感染 vs 0-未感染;

#### v3. 截距系数,取1;

#### v4. 年龄: 真实年龄(月数)-36;

#### v5. 眼睛是否干涩: 1-是 vs 0-否;

#### v6. 季节的正弦: sin(visit);

#### v7. 季节的余弦: cos(visit);

#### v8. 性别: 1-女性 vs 0-男性;

#### v9. 身高;

#### v10. 是否头晕: 1-是 vs 0-否;

#### v11. 第k次观测: k=1-6;

#### v12. baseline age;

#### v13. 季节指标: 1-春,2-夏,3-秋,4-冬;

#### v14. k*k

#### *************************************************************

#### 建模--响应变量为v2,协变量-v5+v6+v7+v8+v9+v10,非参协变量-v12

#### *************************************************************

#### split(y,x) -->将数据集y按x分组

###################################################################

library(MASS)

library(splines)

dat<-read.table("indon.dat",header=FALSE)

dat<-as.matrix(dat)

np<-ncol(dat)

id<-dat[,1]

obs<-lapply(split(id,id),"length")

obs<-as.numeric(obs)

nobs<-max(obs)

temp<-split(dat,id)

nsub<-length(temp)

time_start=Sys.time()

################################################################

####### 计算初值 ####

##### ************************************************* ####

##### 在组内独立假定下求初值,这是因为根据GEE的理论结果, ##

##### 这样得到的初值仍是根方相合的. ##

##### 在组内独立假定下,直接用广义线性模型的估计方法 ##

##### 就可以得到相关参数的估计. ##

##### ************************************************** ##

##### bu-->非参函数在各点的的样条基构成的矩阵,注意 ##

##### 所用的bs命令得到的样条基是不含截距项的. ##

##### tet.ini-->参数beta和样条基系数gamma的初值. ##

##### 注意tet.ini中含截距项. ##

##############################################################

y<- dat[,2]

x<- dat[,5:10]

u<- dat[,12]

bu<- bs(u,df=6)

tet.ini<- scan(file="RealDateAllData.txt")

#################################################################

#### n--> 样本量 ##

#### m--> 样本重复观测次数 ##

#### va--> 样本标准差 ##

#### L--> B样条基的阶数,取L=6的3次B样条 ##

#### np--> 参数的个数+1(截距项的维数) ##

#### npe--> 对非参函数作样条逼近后,待估参数的个数 ##

#### nb--> 基矩阵中的矩阵的个数,对CS结构,nb=2 ##

#### ne--> 估计方程个数=npxnb ##

#### M1--> CS结构对应的基矩阵之一,为单位阵 ##

#### M2--> CS结构对应的基矩阵之二,对角元为零, ##

#### 非对角元均为1. ##

#### ***************************************************** ##

#### 以下为预定义的初值 ##

#### ***************************************************** ##

#### PB --> 伪设计阵,由截距、X和样条基构成 ##

#### gi --> 广义score向量, dx1维 ##

#### dgi--> gi的一阶偏导数矩阵, dxp维 ##

#### dQn1-> 估计函数Qn的一阶偏导数的第一部分, p维 ##

#### tet.hat--> 全部参数的估计值 ##

#### it_max--> 求根时的迭代次数上限 ##

#### tolerance--> 判断所得估计是否收敛的阈值 ##

#################################################################

#################################################################

lambda=exp(seq(-15,5,length.out=50))

n<-nsub

N=length(id)

L=6

p<-6

p1<-p+1

p2=1

np<- p1+L*p2

nbm=2

ne<-np*nbm

it_max<-500

tolerance<-1e-7

fails=0

################################################################

###### QIF-SCAD的牛顿迭代求解 ##########

#### ************************************************** ####

#### index-->取0表示迭代发散,未得到解. ####

#### itmax-->最大迭代次数 ####

#### k--> 迭代次数,初值为1 ####

#### dif--> 前后两次估计值的差 ####

#### norm--> 前后两次估计值的差的二范数 ####

#### tolerance-->精度要求 ####

#### index0--> 被估为零的参数分量所在位置

#### index--> 估计值不为零的参数分量所在的位置

#### df_lam--> 估计值不为零的参数分量的个数

#### Qn_p--> 惩罚QIF函数

#### bic_lam--> 给定lambda下模型的BIC值

#### ic.index--> 给定lambda下SCAD估计中零与非零分量的位置

#### "1"表示非零分量,"0"表示零分量.

################################################################

bic<-65536

bic.scad=0

for (j in 1:50)

{

lam<-lambda[j]

index=0

index1=c(1:p)

k=1

tet<-tet.ini

while (k<=it_max){

tet1<-tet

X1<-if (length(index1)!=0) x[,index1] else rep(0,N)

tet<-tet-renew(tet)

len<-length(tet)

index0<-which(abs(tet[-c(1,(len-L+1):len)])<0.01)+1

if (length(index0)!=0) tet[index0]<-0

norm<-sqrt(t(tet-tet1)%*%(tet-tet1))

index10<-which(tet[-c(1,(len-L+1):len)]!=0)

index1<-index1[index10]

if (length(index0)!=0) tet<-tet[-index0]

if (norm<tolerance) { index<-1

len<-length(tet)

temp<-rep(0,np)

temp[1]<-tet[1]

temp[(index1+1)]<-tet[-c(1,(len-L+1):len)]

temp[(np-L+1):np]<-tet[(len-L+1):len]

tet.lam<-temp

break}

k<-k+1

}

if (index==0) fails<-fails+1

df_lam<-length(which(abs(tet.lam[2:10])>1e-7))

Qn_p<-Qn(tet.lam)+p_scad(tet.lam,lam)

bic_scad<-Qn_p+df_lam*log(nsub)

bic.scad[j]<-bic_scad

temp<-rep(0,np)

if (bic_scad<bic){

bic<-bic_scad

tet.scad<-tet.lam

temp[which(abs(tet.lam)>1e-7)]<-1

ic.scad.index<-temp

}

}

######################################################################

###### 输 出 结 果 ######

###### *********************************************** ######

###### 程序耗时 9.83seconds ######

################################################################

time_end=Sys.time()

time_end-time_start

write(tet.scad,file="RealDataScad.txt")

intercept.scad<-tet.scad[1]

bta.scad<-tet.scad[2:p1]

gama.scad<-tet.scad[(p1+1):np]

fun1<-c("QIF Estimator")

fun2<-c("DIF-SCAD Estimator")

u<-runif(300,-32,40)

u<-sort(u)

buu<-bs(u,df=6)

intercept.hat<-tet.ini[1]

gama.hat<-tet.ini[(p1+1):np]

yhat<-intercept.hat+buu%*%gama.hat

yyhat<-intercept.scad+buu%*%gama.scad

xt<-u/12+3

pdf("CurveCompare.pdf")

plot(xt,yhat,type="l",lty=1,col="black",xlim=c(0,7),ylim=c(-8,0),xlab="Baseline age (years)",lwd=2,ylab="f(age)")

text(3.63,-2.3,"f(age)")

lines(xt,yyhat,lty=2,col="red")

legend("topright", c(fun1,fun2),lty=c(1,2),col=c("black","red"))

dev.off()

**real-scad.R**: main program of QIF-SCAD under GPLVM for the real data, while the degree of freedom of the model is defined as Xue, Qu and Zhou [9]; The result is saved in RealDataScad-xue.txt.

###################################################################

##### 纵向数据广义部分线性变系数模型 SCAD 惩罚变量选择 rev1.1

##### 实例分析--呼吸道感染数据

##### 数据来源--https://www.hsph.harvard.edu/xlin/data.html

###################################################################

#### id --> 个体的编号,个体按编号排序,不同的ID对应不同的个体

#### np --> 所研究的数据集包含的变量个数

#### obs --> 向量,每个分量表示相应个体的观测次数

#### nobs--> 个体的最大观测次数.

#### delobs--> 观测次数少于nobs的的个体的ID对应的位置

#### temp --> 数据按ID分组

#### nsub --> 数据所包含的个体总数

#### *************************************************************

#### loc1 --> 第i个个体第一次观测所在的行号

#### loc2 --> 第i个个体最后一次观测所在的行号

#### *************************************************************

#### indon数据一共14列,从左至右的14个指标分别是--

#### v1. 不同个体的识别ID;

#### v2. 呼吸道是否感染: 1-感染 vs 0-未感染;

#### v3. 截距系数,取1;

#### v4. 年龄: 真实年龄(月数)-36;

#### v5. 眼睛是否干涩: 1-是 vs 0-否;

#### v6. 季节的正弦: sin(visit);

#### v7. 季节的余弦: cos(visit);

#### v8. 性别: 1-女性 vs 0-男性;

#### v9. 身高;

#### v10. 是否头晕: 1-是 vs 0-否;

#### v11. 第k次观测: k=1-6;

#### v12. baseline age;

#### v13. 季节指标: 1-春,2-夏,3-秋,4-冬;

#### v14. k*k

#### *************************************************************

#### 建模--响应变量为v2,协变量-v5+v6+v7+v8+v9+v10,非参协变量-v12

#### *************************************************************

#### split(y,x) -->将数据集y按x分组

###################################################################

rm(list=ls())

library(MASS)

library(splines)

source("realgains.R")

dat<-read.table("indon.dat",header=FALSE)

dat<-as.matrix(dat)

np<-ncol(dat)

id<-dat[,1]

obs<-lapply(split(id,id),"length")

obs<-as.numeric(obs)

nobs<-max(obs)

temp<-split(dat,id)

nsub<-length(temp)

##############################################################

###### 惩罚调节参数格子点

##############################################################

lambda=exp(seq(-10,8,length.out=50))

nlamda<-length(lambda)

np=7 ### 6 个协变量 + 截距

nq=2 ### 年龄以非参形式引入

L=6 ### 样条基的维数

nac<-np+nq*L ### 样条化后全部待估参数个数

nm<-2 ### 基矩阵个数

ne<-nac*nm ### 估计方程总数

it_max=500 ### 牛顿迭代的次数上限

M1<- diag(nobs) ### CS结构的两个基矩阵之一

M2<- 1*(row(M1)!=col(M1)) ### CS结构的两个基矩阵之二

tolerance<-1e-7

grids=600

hbu<- seq(0,6,length.out=301)[-1] ## 计算 H 范数时在 [0,5] 上取的格子点

time_start=Sys.time()

################################################################

####### 计算初值 ####

##### ************************************************* ####

##### 在组内独立假定下求初值,这是因为根据GEE的理论结果, ##

##### 这样得到的初值仍是根方相合的.

##############################################################

y<- dat[,2]

x<- dat[,5:10]

z<-dat[,9]^2

u<- dat[,12]

uu<- bs(u,df=L)

zu<-z*uu

bu<-cbind(zu,uu)

N=length(y)

tet.ini<- glm(formula=y~x+bu,family=binomial)$coefficients

tet.ini<- as.vector(tet.ini)

##############################################################

###### SCAD 惩罚迭代求解

##############################################################

x<- dat[,4:10]

x[,1]<-1

bic<-65536

fails<-0

for (j in 1:nlamda) {

lam<-lambda[j]

tet<-tet.ini

index=0

ks=1

while (ks<=it_max){

tet1<-tet

tet<-tet-gains(tet)

norm<-sqrt(crossprod(tet-tet1))

if (norm<tolerance) { index<-1

tet.lam<-as.vector(tet)

tet.lam<-mytet(tet.lam)

break }

ks<-ks+1

} #### while 循环结束

if (index!=0) { df.lam<- dfx(tet.lam) ### 薛兰的自由度

penalty<- p.scad(tet.lam,lam)

bic_scad<-Qn(tet.lam)+nsub*penalty+log(nsub)*df.lam

if (bic_scad<bic) {bic<-bic_scad

minlam<-lam

tet.scad<-tet.lam }

}else {fails<-fails+1}

} #### QIF-SCAD 选择结束

significant<-which(abs(tet.lam)[2:np]>1e-7)

##############################################################

###### 求解结束,输出结果, T530耗时 3.657166分钟

##############################################################

time_end=Sys.time()

time_end-time_start

write(tet.scad,file="RealdataSCAD-xue.txt")

**real-scad-tian.R :** **main program of QIF-SCAD under GPLVM for the real data, while the degree of fredom of the model is defined as Tian,Xue and Liu. [15]; The result is saved in RealDateScad-tian.txt**

###################################################################

##### 纵向数据广义部分线性模型的部分 SCAD 惩罚变量选择 rev1.1

##### 实例分析--呼吸道感染数据

##### 数据来源--https://www.hsph.harvard.edu/xlin/data.html

###################################################################

#### id --> 个体的编号,个体按编号排序,不同的ID对应不同的个体

#### np --> 所研究的数据集包含的变量个数

#### obs --> 向量,每个分量表示相应个体的观测次数

#### nobs--> 个体的最大观测次数.

#### delobs--> 观测次数少于nobs的的个体的ID对应的位置

#### temp --> 数据按ID分组

#### nsub --> 数据所包含的个体总数

#### *************************************************************

#### loc1 --> 第i个个体第一次观测所在的行号

#### loc2 --> 第i个个体最后一次观测所在的行号

#### *************************************************************

#### indon数据一共14列,从左至右的14个指标分别是--

#### v1. 不同个体的识别ID;

#### v2. 呼吸道是否感染: 1-感染 vs 0-未感染;

#### v3. 截距系数,取1;

#### v4. 年龄: 真实年龄(月数)-36;

#### v5. 眼睛是否干涩: 1-是 vs 0-否;

#### v6. 季节的正弦: sin(visit);

#### v7. 季节的余弦: cos(visit);

#### v8. 性别: 1-女性 vs 0-男性;

#### v9. 身高;

#### v10. 是否头晕: 1-是 vs 0-否;

#### v11. 第k次观测: k=1-6;

#### v12. baseline age;

#### v13. 季节指标: 1-春,2-夏,3-秋,4-冬;

#### v14. k*k

#### *************************************************************

#### 建模--响应变量为v2,协变量-v5+v6+v7+v8+v9+v10,非参协变量-v12

#### *************************************************************

#### split(y,x) -->将数据集y按x分组

###################################################################

rm(list=ls())

library(MASS)

library(splines)

source("realgains.R")

dat<-read.table("indon.dat",header=FALSE)

dat<-as.matrix(dat)

np<-ncol(dat)

id<-dat[,1]

obs<-lapply(split(id,id),"length")

obs<-as.numeric(obs)

nobs<-max(obs)

temp<-split(dat,id)

nsub<-length(temp)

##############################################################

###### 惩罚调节参数格子点

##############################################################

lambda=exp(seq(-10,8,length.out=50))

nlamda<-length(lambda)

np=7 ### 6 个协变量 + 截距

nq=2 ### 年龄以非参形式引入

L=6 ### 样条基的维数

nac<-np+nq*L ### 样条化后全部待估参数个数

nm<-2 ### 基矩阵个数

ne<-nac*nm ### 估计方程总数

it_max=500 ### 牛顿迭代的次数上限

M1<- diag(nobs) ### CS结构的两个基矩阵之一

M2<- 1*(row(M1)!=col(M1)) ### CS结构的两个基矩阵之二

tolerance<-1e-7

grids=600

hbu<- seq(0,6,length.out=301)[-1] ## 计算 H 范数时在 [0,5] 上取的格子点

time_start=Sys.time()

################################################################

####### 计算初值 ####

##### ************************************************* ####

##### 在组内独立假定下求初值,这是因为根据GEE的理论结果, ##

##### 这样得到的初值仍是根方相合的.

##############################################################

y<- dat[,2]

x<- dat[,5:10]

z<-dat[,9]^2

u<- dat[,12]

uu<- bs(u,df=L)

zu<-z*uu

bu<-cbind(zu,uu)

N=length(y)

tet.ini<- glm(formula=y~x+bu,family=binomial)$coefficients

tet.ini<- as.vector(tet.ini)

##############################################################

###### SCAD 惩罚迭代求解

##############################################################

x<- dat[,4:10]

x[,1]<-1

bic<-65536

fails<-0

for (j in 1:nlamda) {

lam<-lambda[j]

tet<-tet.ini

index=0

ks=1

while (ks<=it_max){

tet1<-tet

tet<-tet-gains(tet)

norm<-sqrt(crossprod(tet-tet1))

if (norm<tolerance) { index<-1

tet.lam<-as.vector(tet)

tet.lam<-mytet(tet.lam)

break }

ks<-ks+1

} #### while 循环结束

if (index!=0) { df.lam<- dfm(tet.lam) ### 田瑞琴自由度

penalty<- p.scad(tet.lam,lam)

bic_scad<-Qn(tet.lam)+nsub*penalty+log(nsub)*df.lam

if (bic_scad<bic) {bic<-bic_scad

minlam<-lam

tet.scad<-tet.lam }

}else {fails<-fails+1}

} #### QIF-SCAD 选择结束

significant<-which(abs(tet.lam)[2:np]>1e-7)

##############################################################

###### 求解结束,输出结果, T530耗时 3.631946分钟

##############################################################

time_end=Sys.time()

time_end-time_start

write(tet.scad,file="RealdataSCAD-tian.txt")

**realgains.R** : **all sub-program needed for real-scad.r and real-scad-tian.r.**

#####################################################################

####### 不要截距项的 scad 更新

#####################################################################

gains<-function(xt,method="scad"){ ### xt 为每步迭代中 tet 的初值

len<-length(xt)

naci<-len*nm

bta <- xt[1:np]

gama<- xt[-(1:np)]

eta<- x%*%bta+bu%*%gama

mu<- 1/(1+exp(-eta))

dh<-mu*(1-mu)

va<-as.vector(sqrt(dh))

temp<-which(va!=0)

va[temp]<-1/va[temp]

res<- y-mu

omega<- 0

gn<- 0

dgn<- 0

loc2=0

gi<-rep(0,ne)

dgi<-matrix(0,ne,nac)

for (i in 1:nsub) {

m <-obs[i]

loc1<-loc2+1

loc2<-loc2+m

M1<- diag(m)

M2<- 1*(row(M1)!=col(M1))

PB<-matrix(0,m,nac)

PB[,(1:np)]<- x [loc1:loc2,]

PB[,-(1:np)]<- bu [loc1:loc2,]

dmu<- if (loc1==loc2) dh[loc1]*PB else diag(dh[loc1:loc2])%*%PB

gi[1:nac]<- t(dmu)%*%M1%*%res[loc1:loc2]

gi[(nac+1):naci]<- t(dmu)%*%M2%*%res[loc1:loc2]

omega<-omega+gi%*%t(gi)

gn<-gn+gi

dgi[1:nac,]<- t(dmu)%*%M1%*%dmu

dgi[(nac+1):naci,]<- t(dmu)%*%M2%*%dmu

dgn<-dgn+dgi

}

omega.inv<-ginv(omega)

gn<-gn/nsub

dgn<- -dgn/nsub

dQn<-2*t(dgn)%*%omega.inv%*%gn

ddQn<-2*t(dgn)%*%omega.inv%*%dgn

sig.lam<- lam.scad(xt,lam)

ddQn.inv<-ginv(ddQn+nsub*sig.lam)

temp<-ddQn.inv%*%(dQn+nsub*sig.lam%*%tet)

temp<-as.vector(temp)

return(temp)

}

#################################################################

### 二次推断函数 Qn(tet)

#################################################################

Qn<-function(xt) {

len<-length(xt)

naci<-len*nm

bta<- xt[1:np]

gama<- xt[-(1:np)]

eta<- x%*%bta+bu%*%gama

mu<- 1/(1+exp(-eta))

dh<-mu*(1-mu)

va<-as.vector(sqrt(dh))

temp<-which(va!=0)

va[temp]<-1/va[temp]

res<- y-mu

omega<- 0

gn<- 0

dgn<- 0

gi<-rep(0,ne)

dgi<-matrix(0,ne,nac)

loc2<-0

for (i in 1:nsub) {

m<-obs[i]

loc1<-loc2+1

loc2<-loc2+m

M1<- diag(m)

M2<- 1*(row(M1)!=col(M1))

PB<-matrix(0,m,nac)

PB[,1:np]<- x[loc1:loc2,]

PB[,(np+1):nac]<- bu[loc1:loc2,]

dmu<- if (loc1==loc2) dh[loc1]*PB else diag(dh[loc1:loc2])%*%PB

gi[1:nac]<- t(PB)%*%M1%*%res[loc1:loc2]

gi[(nac+1):naci]<- t(PB)%*%M2%*%res[loc1:loc2]

omega<-omega+gi%*%t(gi)

gn<-gn+gi

}

omega<-omega/nsub

omega.inv<-ginv(omega)

gn<-gn/nsub

temp<-nsub*t(gn)%*%omega.inv%*%gn

temp<-as.numeric(temp)

return(temp)

}

#################################################################

### SCAD 惩罚迭代式中的惩罚矩阵 Sig_lam

#################################################################

lam.scad<-function(xt,lamda,a=3.7){

dimt<-length(xt)

temp<-diag(dimt)

locb<-1:np

fun<-xt[locb]

weight<-rever(fun)

value<-sapply(fun,d.scad)*weight

temp[locb,locb]<- diag(value)

fun<-xt[-locb]

fun<-matrix(fun,nr=L)

Hnorm<-apply(fun,2,hnorm)

len<-length(Hnorm)

weight<-rever(Hnorm)

value<-sapply(Hnorm,d.scad)*weight

bsu<-bs(hbu,df=L)

Hscad<-t(bsu)%*%bsu/grids

if (len>1) {temp[-locb,-locb]<- diag(value)%x%Hscad

}else{ temp[-locb,-locb]<- value*Hscad }

return(temp)

}

#################################################################

######### 计算样条向量的 H 范数

#################################################################

hnorm<-function(gt,grids=400){ ### gt 为样条系数向量

grids<-300

hbu<- seq(0,1,length.out=grids+1)[-1]

bsu<-bs(hbu,df=L)

temp<-sqrt(crossprod(bsu%*%gt)/grids)

return(temp)

}

#################################################

########## 惩罚函数中的倒数加权向量

##################################################

rever<-function(xt){ ## 对向量 xt 的绝对值向量取倒数

temp<- abs(xt) ## 0元不变

temp[which(temp<1e-36)]<-0

index<- which (temp!=0)

temp[index]<- 1/temp[index]

return(temp)

}

##############################################################

### scad 惩罚函数形式

##############################################################

scad<-function(xt,lamda=lam,a=3.7){

if(abs(xt)< lamda) return (lamda*abs(xt))

else if (abs(xt)<=a*lamda) return ((xt^2+lamda^2 -2*a*lamda*abs(xt))/(2-2*a))

else return ((a+1)^2*lamda^2/2)

}

#################################################

########## SCAD 惩罚函数的导数 (对标量)

##################################################

d.scad<-function(xt,lamda=lam){

a<-3.7

temp<-(a*lamda-abs(xt))/(a-1)

if (abs(xt)<=lamda) temp<- lamda

if (abs(xt)>=a*lamda) temp<- 0

return(temp)

}

#################################################################################

#### 对 theta 的 的SCAD 惩罚

#################################################################################

p.scad<-function(xt,lamda=lam){ ## xt 为 theta 的估计

bta<-xt[1:np]

bta.scad<-sapply(bta,scad)

gama<-xt[-(1:np)] ### 样条系数向量组

gama<-matrix(gama,L,nq) ### 将向量组转变为 nq 个 L 维向量组

gam.norm<-apply(gama,2,hnorm) ### 计算各向量组的 H 范数

gam.scad<-sapply(gam.norm,scad) ### 各向量组的 SCAD 惩罚

temp<-sum(bta.scad)+sum(gam.scad) ### 对 theta 的惩罚

return(temp)

}

#################################################################################

#### 模型自由度 dfx: 薛兰等定义的自由度

#### 模型自由度 dfm: 通常定义的自由度

#################################################################################

dfm<-function(xt){

btat<- xt[1:np]

df.bta<- sum(btat!=0)

gmat<- xt[-(1:np)]

gmat<- abs(matrix(gmat,nr=L))

temp<- apply(gmat,2,sum)

df.gam<- sum(temp!=0)

the.df<-df.bta+df.gam

return(the.df)

}

#################################################################################

dfx<-function(xt) {

len<-length(xt)

naci<-len*nm

bta<- xt[1:np]

gama<- xt[-(1:np)]

eta<- x%*%bta+bu%*%gama

mu<- 1/(1+exp(-eta))

dh<-mu*(1-mu)

va<-as.vector(sqrt(dh))

temp<-which(va!=0)

va[temp]<-1/va[temp]

res<- y-mu

omega<- 0

gn<- 0

dgn<- 0

PB<-matrix(0,nobs,len)

gi<-rep(0,naci)

dgi<-matrix(0,naci,len)

loc2<-0

for (i in 1:nsub) {

m <-obs[i]

loc1<-loc2+1

loc2<-loc2+m

M1<- diag(m)

M2<- 1*(row(M1)!=col(M1))

PB<-matrix(0,m,nac)

PB[,(1:np)]<- x [loc1:loc2,]

PB[,-(1:np)]<- bu [loc1:loc2,]

dmu<- if (loc1==loc2) dh[loc1]*PB else diag(dh[loc1:loc2])%*%PB

gi[1:nac]<- t(dmu)%*%M1%*%res[loc1:loc2]

gi[(nac+1):naci]<- t(dmu)%*%M2%*%res[loc1:loc2]

omega<-omega+gi%*%t(gi)

gn<-gn+gi

dgi[1:nac,]<- t(dmu)%*%M1%*%dmu

dgi[(nac+1):naci,]<- t(dmu)%*%M2%*%dmu

dgn<-dgn+dgi

}

omega<-omega/nsub

omega.inv<-ginv(omega)

gn<-gn/nsub

dgn<- -dgn/nsub

dQn<-2*t(dgn)%*%omega.inv%*%gn ### 二次推断函数 Qn 的一阶导

ddQn<-2*t(dgn)%*%omega.inv%*%dgn ### 二次推断函数 Qn 的二阶导

ddQn.inv<-ginv(ddQn+nsub*lam.scad(xt,lam))

temp<-sum(diag(ddQn.inv%*%ddQn))

return(temp)

}

###################################################################

#### 按给定阈值 1e^-6 决定 bta_i 或 gamma_i 是否为零

###################################################################

mytet<-function(xt){

bta<- xt[1:np]

index0<-which(abs(bta)<1e-6)

bta[index0]<-0

gama<-xt[-(1:np)]

gama<-matrix(gama,nr=L)

Hnorm<-apply(gama,2,hnorm)

index0<-which(Hnorm<1e-6)

gama[,index0]<-0

gama<-as.vector(gama)

temp<-c(bta,gama)

return(temp)

}

**funcompare.R : the program to plot the estimated curve in 3 setup: GPLM with no penalty, GPLM with SCAD penalty and GPLVM with SCAD penalty.**

###########################################################

##### 纵向数据广义部分线性变系数模型的部分 SCAD 惩罚变量选择 rev1.1

##### 实例分析--呼吸道感染数据的年龄函数的对比估计

##### 数据来源--https://www.hsph.harvard.edu/xlin/data.html

###################################################################

library(MASS)

library(splines)

u<-seq(-32,40,length.out=300)

buu<-bs(u,df=6)

xt<-u/12+3

######################################################################

###### QIF Estimator for alpha(u) in GPLM

#####################################################################

tet<-scan(file="RealDateAllData.txt")

gama<-tet[8:13]

yhat<-tet[1]+buu%*%gama

######################################################################

###### QIF-SCAD Estimator for alpha(u) in GPLM

#####################################################################

tet.scad1<-scan("RealDataScad.txt")

gama1<-tet.scad1[8:13]

yhat1<-tet.scad1[1]+buu%*%gama1

######################################################################

###### QIF-SCAD Estimator for alpha(u) in GPLVCM

###### the degree of freedom of the model defined by Xue Lan JASA 2010

###### The curve obtained is almost the same with that of Tian JMA 2014

#####################################################################

tet.scad2<-scan("RealdataSCAD-xue.txt")

gama2<-tet.scad2[14:19]

yhat2<-tet.scad2[1]+buu%*%gama2

######################################################################

###### QIF-SCAD 估计, 模型自由度定义同田瑞琴等

#####################################################################

tet.scad3<-scan("RealdataSCAD-tian.txt")

gama3<-tet.scad2[14:19]

yhat3<-tet.scad2[1]+buu%*%gama2

######################################################################

###### 图例文字

#####################################################################

fun1<-"QIF estimator under GPLM"

fun2<-"QIF-SCAD estimator under GPLM"

fun3<-"QIF-SCAD estimator under GPLVCM"

plot(xt,yhat,type="l",lty=1,col="black",xlim=c(0,7),ylim=c(-10,1),xlab="Baseline age (years)",lwd=1,ylab="The estimated curve of f(age)")

lines(xt,yhat1,lty=3,col="red",lwd=1)

lines(xt,yhat2,lty=5,col="blue",lwd=1)

legend("topright",c(fun1,fun2,fun3),lty=c(1,2,6),lwd=c(1,2,1),col=c("black","red","blue"))
